# Sympathetic nervous system-mediated fibro-adipogenic progenitor mobilization drives stroke-related sarcopenia

**DOI:** 10.1038/s41421-026-00899-0

**Published:** 2026-07-07

**Authors:** Yinong Huang, Yilin Liu, Ruijie Li, Mingming Fan, Yixuan Liu, Yiling Wang, Xin Sui, Xiaofeng Yuan, Qiying Lu, Yuan Qiu, Ruijun Li, Jierui Chen, Bingjun Zhang, Sanxin Liu, Chuyun Ou, Yuanchen Ma, Xiaofan Lai, Jie Ren, Zhengqi Lu, Huimin Yi, Weijun Huang, Jiancheng Wang, Yanbing Li, Haipeng Xiao, Andy Peng Xiang

**Affiliations:** 1 Department of Endocrinology, The First Affiliated Hospital of Sun Yat-sen University, Guangzhou, Guangdong China; 2 Center for Stem Cell Biology and Tissue Engineering, Key Laboratory for Stem Cells and Tissue Engineering, Ministry of Education, Sun Yat-sen University, Guangzhou, Guangdong China; 3 National-Local Joint Engineering Research Center for Stem Cells and Regenerative Medicine, Zhongshan School of Medicine, Sun Yat-sen University, Guangzhou, Guangdong China; 4 Department of Critical Care Medicine, the First Affiliated Hospital of Sun Yat-sen University, Guangzhou, Guangdong China; 5 Department of Surgery Intensive Care Unit, The Third Affiliated Hospital of Sun Yat-sen University, Guangzhou, Guangdong China; 6 Department of General Intensive Care Unit, Lingnan Hospital, The Third Affiliated Hospital of Sun Yat-sen University, Guangzhou, Guangdong China; 7 Department of Rehabilitation Medicine, The Third Affiliated Hospital, Sun Yat-sen University, Guangzhou, Guangdong China; 8 Department of Neurology, The Third Affiliated Hospital of Sun Yat-sen University, Guangzhou, Guangdong China; 9 Department of Gastrointestinal Surgery, The First Affiliated Hospital of Sun Yat-sen University, Guangzhou, Guangdong China; 10 Department of Anesthesiology, The First Affiliated Hospital of Sun Yat-sen University, Guangzhou, Guangdong China; 11 Department of Medical Ultrasonics, The Third Affiliated Hospital of Sun Yat-sen University, Guangzhou Guangdong, China; 12 Scientific Research Center, The Seventh Affiliated Hospital of Sun Yat-sen University, Shenzhen, Guangdong China; 13 Department of Histoembryology and Cell Biology, Zhongshan School of Medicine, Sun Yat-sen University, Guangzhou, Guangdong China

**Keywords:** Mesenchymal stem cells, Mechanisms of disease

## Abstract

Patients who survive stroke usually experience rapid muscle wasting and an increased risk of physical disability. Although multifactorial interactions, including malnutrition, disuse, systemic catabolic imbalance, and neurohormonal dysregulation, are thought to contribute to the progression of stroke-related sarcopenia, the underlying mechanisms of this brain–muscle crosstalk remain elusive. Muscle-resident fibro-adipogenic progenitors (FAPs) are indispensable for maintaining muscle homeostasis and function as initial sensors of external perturbations. In the present study, we report that FAPs rapidly respond to the overactive sympathetic nervous system (SNS) and egress from the muscle niche into circulation during the acute phase of stroke. FAP-specific ablation of adrenoceptor beta 2 (Adrb2) markedly ameliorated stroke-related sarcopenia, highlighting the central role of SNS-mediated FAP loss in its pathogenesis. Mechanistically, increased norepinephrine release initiates FAP mobilization through the activation of pro-migratory signals and the degradation of extracellular matrix components. Using transcriptomic profiling, we further characterized insulin growth factor-1 (IGF-1) as a key anti-atrophic executive factor predominantly derived from FAPs. Collectively, our work demonstrates that the SNS-mediated loss of FAPs and subsequent compromised IGF-1 secretion contribute to sarcopenia in mice following stroke. Targeting this mechanism by early anti-sympathetic treatment with propranolol may effectively restore muscle homeostasis and mass after stroke.

## Introduction

Stroke is the leading cause of long-term physical disability in adults, resulting in a tremendous socioeconomic burden. Even after receiving prompt treatment, ~30% of stroke patients remain unable to walk without assistance^1^. Skeletal muscle is the main effector organ responsible for impaired mobility after cerebral injury. During the early stage of stroke, patients experience a particularly rapid loss of muscle mass and critical deterioration of muscle function in both paretic and non-paretic limbs, which is referred to as stroke-related sarcopenia^2^. With an estimated prevalence of 42%, stroke-related sarcopenia is among the most frequent complications observed in stroke survivors^3^. In contrast to the extensively explored local neurovascular defects, the cellular and molecular mechanisms involved in skeletal muscle dysregulation caused by stroke are underexplored.

A combination of confounding factors, including malnutrition, disability, inflammation and overactivated catabolic signaling, have been proposed to underlie stroke-related sarcopenia^4^. However, both reduced locomotor activity and decreased food intake have been reported to be temporal and transient in experimental stroke mice, indicating that disuse and impaired feeding may not be major factors responsible for stroke-related sarcopenia^5–7^. Furthermore, structural and functional deterioration occurring in bilateral (instead of contralateral only) skeletal muscle suggests that some systemic factors, such as stress hormones, may play a predominant role in this process. Acute cerebral ischemia with extended brain lesions triggers a global stress response mediated by overactivation of the sympathetic nervous system (SNS) and subsequent catecholamine overflow^8,9^. Recent evidence suggests that skeletal muscle degradation may be a main target of the overall increase in sympathetic and catabolic signaling after stroke^10,11^. Nevertheless, the cellular mechanism of muscle homeostatic disturbance caused by SNS overactivation after stroke and the downstream signaling cascade in myofibers remain elusive.

The maintenance of skeletal muscle homeostasis requires the fine coordination of different cellular components in the muscular niche, especially tissue-resident stem cells^12^. With their ability to proliferate and regenerate damaged myofibers, PAX7^+^ muscle satellite cells (MuSCs) are recognized as the direct executors of muscle regeneration following muscle injury^13^. However, Fry et al. demonstrated that inducible ablation of MuSCs does not accelerate or exacerbate sarcopenia in aged mice, indicating that MuSCs may not be globally required for maintaining myofiber volume under homeostatic conditions^14^. In contrast, emerging evidence suggests that a population of interstitial fibro-adipogenic progenitors (FAPs) is pivotal for homeostatic muscle maintenance because it provides a favorable and trophic environment for myofibers as well as MuSCs^15,16^. These non-myogenic progenitors can be broadly identified as mesenchymal progenitors because of their expression of surface markers, including platelet-derived growth factor receptor alpha (PDGFRα), stem cell antigen-1 (SCA-1), CD90, and CD34^17^. Recent studies have revealed the absolute requirement of FAPs for maintaining both the MuSC pool and skeletal muscle architecture through the genetic depletion of fibroblast activation protein-α^+^ or PDGFRα^+^ FAPs^18,19^. Under homeostatic conditions, FAP-ablated mice exhibit muscle atrophy and MuSC dysfunction^19^, indicating that FAP disruption can directly contribute to muscle wasting. On the basis of these findings, these muscle interstitial cells are increasingly considered indispensable for the pathogenesis of muscle disorders. To date, no study has extensively explored FAP alterations under SNS innervation or its influence on stroke-related sarcopenia.

In the present study, we demonstrate that overactive SNS-mediated FAP egress from skeletal muscle induced stroke-related sarcopenia in a murine middle cerebral artery occlusion (MCAO) model. In addition, the results of preclinical experiments showed that early administration of the β-adrenergic receptor antagonist propranolol partially prevented FAP migration and muscle wasting in stroke mice. Mechanistically, we report that FAP loss culminates in enhanced catabolism and disruption of muscle homeostasis through impaired insulin growth factor-1 (IGF-1) signaling.

## Results

### MCAO induces early sarcopenia and FAP loss

**Fig. 1:**
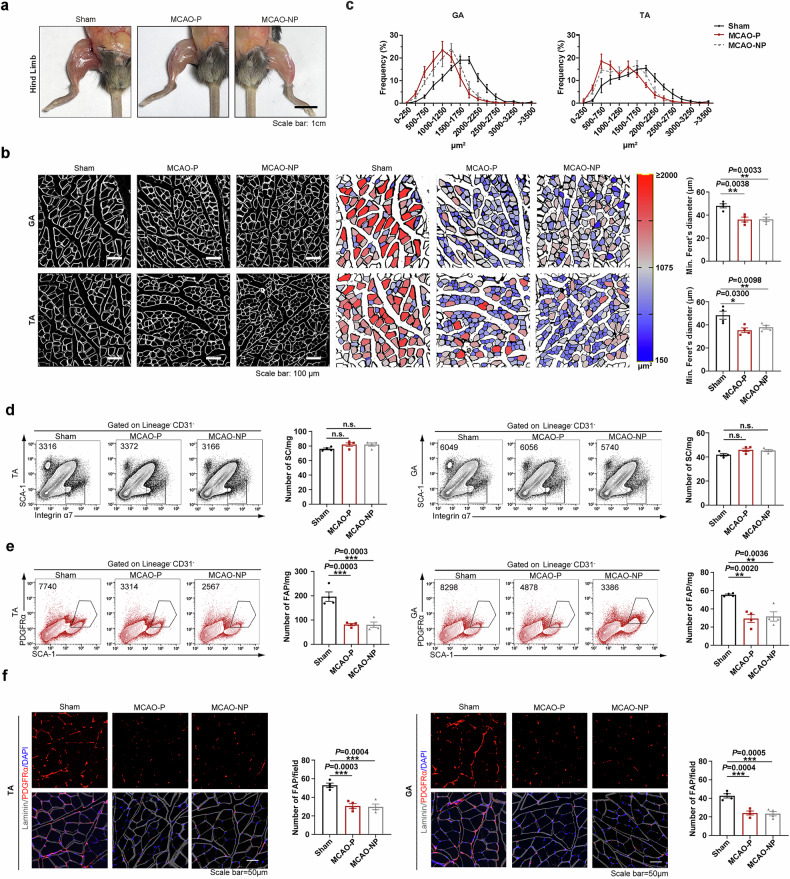
MCAO induces early sarcopenia and FAP loss. **a** Representative images of the lower limbs of pair-fed sham-operated and MCAO mice 3 days after stroke. **b** Immunostaining of TA and GA muscle for laminin and quantification of minimal Feret diameter. *n* = 4. **c** Quantification of the CSA frequency of myofibers in the TA and GA muscles. *n* = 4. **d** FACS analysis showing that Lineage^−^CD31^−^SCA-1^−^Integrin α7^+^ SCs from the hindlimbs were not altered after MCAO. *n* = 4. **e** Representative FACS plots and quantified numbers of Lineage^−^CD31^−^PDGFRα^+^SCA-1^+^ FAPs from both paretic and nonparetic skeletal muscles (TA and GA) on post-stroke Day 3. *n* = 4. **f** Representative confocal images of anti-PDGFRα (red) and anti-Laminin (gray) staining in the TA and GA muscles of sham-operated and MCAO mice. *n* = 4.

Male C57BL/6 mice were subjected to 60 min of MCAO and reperfusion and were then observed regularly for body weight alterations (Supplementary Fig. S1a). To limit the confounding influence of malnutrition, both sham-operated and MCAO mice were pair-fed a jelly diet for ease of post-operative digestion and nutrient absorption. Compared with the sham group, cerebral ischemia induced dramatic weight loss, which peaked on Day 3 (19.24% ± 2.46%) after stroke and remained at low levels until the end of the study (Supplementary Fig. S1b). As the major component of body weight, the skeletal muscle mass of the quadriceps (QUA), gastrocnemius (GA), tibialis anterior (TA), and soleus (SOL) significantly decreased in both the paretic (MCAO-P) and non-paretic (MCAO-NP) limbs of MCAO mice (Supplementary Fig. S1c). In line with the reduction in muscle weight, we detected a decrease in the cross-sectional area (CSA) of myofibers in the bilateral limbs following MCAO (Fig. 1a–c).

Given the central role of tissue-resident stem cells in maintaining muscle homeostasis, we first investigated whether MuSCs or FAPs were affected during stroke-related sarcopenia. To address this question, we used flow cytometry to determine the number of Lineage^−^ (CD3^−^, B220^−^, CD11b^−^, Ly6G^−^, TER-119^−^) CD31^−^SCA-1^−^Integrin α7^+^ satellite cells and Lineage^−^CD31^−^PDGFRα^+^SCA-1^+^ FAPs at 3 days after stroke, which is the time point when substantial loss of muscle mass was detected. The number of FAPs isolated from the bilateral hindlimb muscles of MCAO mice was significantly reduced, whereas the MuSC count remained undisturbed after stroke (Fig. 1d, e; Supplementary Fig. S2). Moreover, the number of Lineage^+^ immune cells did not significantly differ from that in the sham-operated control group (data not shown). In accordance with the flow cytometry results, immunostaining revealed a prominent reduction in the number of PDGFRα^+^ FAPs across the TA and GA muscles of MCAO mice (Fig. 1f).

### Stroke mobilizes the egress of FAPs from skeletal muscle into circulation

Given that macrophages are involved in the clearance of FAPs after acute muscle injury^20,21^, we first quantified the co-labeling of PDGFRα with F4/80 for phagocytosis at different time points following MCAO. Immunofluorescence staining revealed no significant increase in macrophage clearance of PDGFRα^+^ FAPs within the first 3 days after MCAO (Supplementary Fig. S3a). Given that proapoptotic executioner caspases have been implicated in stroke-related sarcopenia^6^, we subsequently explored whether FAPs undergo programmed cell death early after stroke by immunostaining for terminal deoxynucleotidyl transferase dUTP nick end labeling (TUNEL) on PDGFRα^+^ FAPs (Supplementary Fig. S3b). Other possible cellular fates, including necrosis and senescence, were examined by co-labeling PDGFRα with RIP3 and p16^INK4a^ from 12 h to 3 days after stroke (Supplementary Fig. S3c, d). Pro-fibrotic and adipogenic differentiation was determined by Masson’s trichrome and Oil Red O staining 7 days after MCAO (Supplementary Fig. S3e–g). Notably, no apparent evidence of apoptosis, necrosis, senescence or terminal differentiation was detected in the FAPs of MCAO mice. Furthermore, bulk RNA sequencing (RNA-seq) analysis of FAPs revealed no remarkable upregulation of the expression of molecules involved in pro-fibrotic (Col1a1, Smad2, and Smad3) or adipogenic signaling (Pparg and Cebpa) induced by MCAO (Supplementary Fig. S3h–i). Notably, no significant alterations in the expression of PDGFRα were detected after MCAO (Supplementary Fig. S3j). On the basis of these findings, we hypothesized that stroke-induced FAP loss is largely due to FAP emigration rather than to cell death, senescence, differentiation, and phagocytosis or a mere reduction in PDGFRα expression in FAPs.

**Fig. 2:**
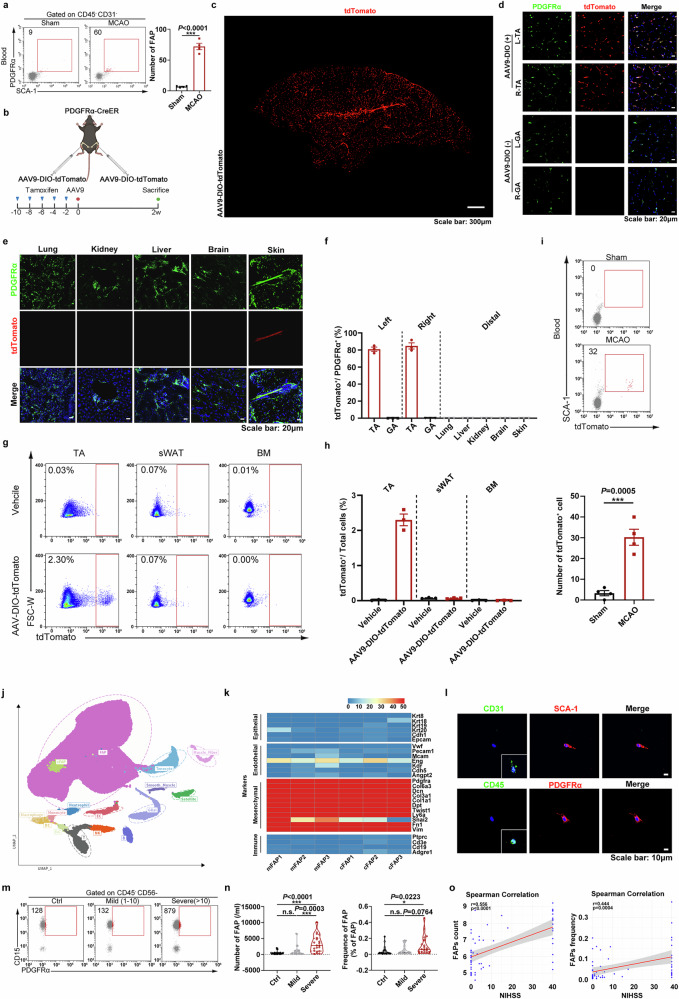
The loss of FAPs is due to the migration of FAPs into circulation. **a** Representative FACS panels for mouse cFAPs defined as CD45^−^CD31^−^PDGFRα^+^SCA-1^+^ cells 3 days after MCAO. *n* = 4. **b** Schematic diagram showing the strategy to specifically label FAPs derived from the TA muscle. **c** Confocal images of entire TA cross-sections showing the efficiency and specificity of Cre-dependent tdTomato expression in PDGFRα^+^ FAPs following the intramuscular injection of AAV9-DIO-tdTomato. **d** Representative images of TA and GA cross-sections showing that PDGFRα-tdTomato-labeled FAPs were distributed in the muscle interstitium, suggesting specific recombination. **e**, **f** Representative fluorescence micrographs (**e**) and corresponding quantitative analysis (**f**) demonstrating the absence of Cre-mediated tdTomato expression in the lung, liver, kidney, brain and skin. **g**, **h** Representative FACS panels (**g**) and quantitative analysis results showing (**h**) the percentage of tdTomato-labeled FAPs in the TA, subcutaneous white adipose tissue (sWAT) and bone marrow (BM) following the intramuscular injection of AAV9-DIO-tdTomato. *n* = 3. **i** Representative FACS panels showing the number of PDGFRα-tdTomato-labeled FAPs derived from the TA muscle in the peripheral blood at 1 day after stroke. *n* = 4. **j** UMAP showing the similarity between cFAPs and mFAPs. **k** Gene expression heatmaps of cFAPs. **l** Immunofluorescence images confirming the mesenchymal lineage (PDGFRα, SCA-1) rather than the endothelial (CD31) or hematopoietic (CD45) lineage of cFAPs. **m**, **n** FACS analysis (**m**) and quantification (**n**) of the number and frequency of cFAPs in both healthy controls and stroke patients. Ctrl *n* = 22, mild *n* = 18, severe *n* = 20. **o** Non-parametric statistical analysis (Spearman correlation) of the association of the FAP count or frequency with the NIHSS score of stroke patients was performed.

Moreover, we hypothesized that the escape of FAPs, rather than cell death, may contribute to stroke-induced FAP loss. We examined peripheral blood and detected an increase in circulating CD45^−^CD31^−^PDGFRα^+^SCA-1^+^ cells in MCAO mice (Fig. 2a), supporting the notion that FAPs egress from skeletal muscle into circulation. To obtain direct evidence of stroke-mediated FAP release from skeletal muscles into circulation, *PDGFRα-CreER* mice were given five intraperitoneal injections of tamoxifen (40 mg/kg, administered every other day) and subsequently underwent an intramuscular injection of AAV9-DIO-tdTomato (5 × 10^10^ total vector genomes) in TA muscle. Two weeks after AAV administration, the mice were anesthetized by isoflurane inhalation and subjected to MCAO surgery (Fig. 2b). To exclude potential systemic leakage, we co-stained tdTomato with PDGFRα in non-injected GA muscle, proximal tissues (i.e., skin, inguinal adipose tissue, and bone marrow) and distant tissues (i.e., lung, liver, kidney, and brain). Immunostaining confirmed the specific co-localization of tdTomato and PDGFRα in TA muscle sections only, with no detectable signal in the GA muscle, lung, liver, kidney, brain, or skin (Fig. 2d–f). Moreover, we examined the expression patterns of tdTomato and PDGFRα in the TA, white adipose tissue, and bone marrow using flow cytometry. Consistently, the co-expression of tdTomato and PDGFRα was detected exclusively in the TA (Fig. 2g, h). Consistent with previous findings^22,23^, our results validate the highly specific transduction of tdTomato in TA-resident FAPs achieved with this model. In line with the results from MCAO mice, an increase in tdTomato-labeled FAPs from TA muscles was detected in peripheral blood one day following stroke (Fig. 2i).

To further confirm the egress of muscle-resident FAPs, we crossed *PDGFRα-CreER* mice with the Cre-mediated tdTomato fluorescence reporter tool strain *Ai9* to label and trace FAPs (Supplementary Fig. S4a). We implemented a local tamoxifen metabolite delivery system to induce CreER activity in bilateral TA muscles and exclude the impact of stromal cells from distant tissues. As previously described^19^, endoxifen (Edx), a tamoxifen metabolite, was impregnated with melted polycaprolactone (PCL) and subcutaneously implanted alongside the anterior surface of the TA muscle. Confocal images taken from whole TA cross-sections illustrated CreER-mediated tdTomato expression in the TA interstitium, with a greater abundance on the anterior side of the TA near the implanted Edx/PCL bar (Supplementary Fig. S4b). Furthermore, a high degree of colocalization between tdTomato fluorescence and immunostaining for PDGFRα expression was detected exclusively in the interstitial space of targeted TA cross-sections (Supplementary Fig. S4c). No appreciable recombination was observed in non-implanted GA muscle, proximal tissues (i.e., inguinal adipose tissue, and bone marrow) or distant tissues (i.e., lung, liver, kidney, and brain), ruling out the possibility of systemic leakage of Edx (Supplementary Fig. S4d–g). We then leveraged this technical system in MCAO mice to test whether tdTomato-labeled FAPs from TA muscles could be detected in peripheral blood following MCAO surgery. In line with the aforementioned findings, we found a significant increase in the number of circulating tdTomato-labeled PDGFRα^+^ FAPs one day after MCAO (Supplementary Fig. S4h). Collectively, these observations substantiate the presence of cFAPs egressed from skeletal muscle.

Next, we investigated the destiny of these cFAPs. We examined multiple tissues, including the brain, lung, spleen, liver, kidney, and bilateral lower limb muscles, from *PDGFRα-CreER* mice intramuscularly injected with AAV9-DIO-tdTomato in the TA muscle and *PDGFRα-CreER;Ai9* reporter mice implanted with Edx/PCL bars. Notably, a noteworthy population of tdTomato-labeled FAPs from the TA muscle could be detected in the lungs of both models, whereas few FAPs were detected in the brain, GA or other tissues at 3 days after MCAO (Supplementary Figs. S4i–j and S5). On the basis of these observations, we hypothesized that the mobilized FAPs migrated through the bloodstream and colonized the lung rather than remaining in prolonged circulation or homing back to the muscle.

In addition, we performed low-input RNA-seq of circulating FAPs (cFAPs) and muscle-resident FAPs (mFAPs) isolated from MCAO mice, the reliability of which was ensured by quality control (Supplementary Fig. S5). Global unbiased analysis of all expressed genes revealed a remarkably high level of similarity in the gene expression profiles between cFAPs and mFAPs (Supplementary Fig. S7a). Unbiased hierarchical clustering revealed similar expression patterns between cFAPs and mFAPs and significant differences among FAPs and other cell types, such as endothelial cells, epithelial cells, monocytes, macrophages, lymphocytes and neutrophils (GSE49910; Supplementary Fig. S7a, b). Furthermore, by projecting cFAP bulk RNA-seq onto the muscle single-cell transcriptome^24^ using projectLSI, we found that cFAPs were assigned to the cluster of muscle-resident FAPs within UMAP (Fig. 2j). In addition, we projected the transcriptional profile of cFAPs onto the characterization of fibroblasts at single-cell resolution across distinct tissues^25^. Notably, uniform manifold approximation and projection (UMAP) revealed an overlap of cFAPs with muscle-resident fibroblasts (Supplementary Fig. S7c). Specifically, both cFAPs and mFAPs predominantly expressed mostly mesenchymal stromal cell (MSC) lineage genes, including Pdgfra and Ly6a (Fig. 2k; Supplementary Fig. S7d). This expression pattern was further validated by immunostaining of in vitro-cultured cFAPs (Fig. 2l). Furthermore, the results of the Gene Ontology (GO) analysis of the differentially expressed genes (DEGs) highlighted the upregulation of migratory gene signature sets in the cFAPs compared with their muscle-resident counterparts (Supplementary Fig. S7e). Overall, these results revealed a similarity in the transcriptional patterns among cFAPs and mFAPs, while the mobilized FAPs in blood exhibited a heightened migratory phenotype.

PDGFRα is a well-established pan-stromal cell marker that is also expressed in perivascular cells (pericytes and vascular smooth muscle cells) of various tissues^26–28^. Utilizing recently published single-cell RNA-seq data from skeletal muscle^24^, we found that muscle-resident PDGFRα^+^ cells comprised a small cluster of αSMA^+^ vascular smooth muscle cells (VSMCs) and some tenocytes (Supplementary Fig. S8a, b). Since most tenocytes could be excluded during the preparation of single-cell suspensions for fluorescence-activated cell sorting (FACS), we sought to rule out the confounding effects of perivascular cells. We identified an alternative surface marker of PDGFRα^+^ FAPs, PI16, which was co-expressed with PDGFRα and lightly expressed in αSMA^+^ VSMCs (Supplementary Fig. S8c, d). To further corroborate the observation of stroke-induced FAP egress, we crossed *Pi16-CreERT2* mice with *Ai9* mice and leveraged the aforementioned Edx/PCL model to locally label and trace PI16^+^ FAPs derived from the TA muscle. Consistent with our previous findings, the number of muscle-resident Lineage^−^CD31^−^PI16^+^ FAPs was substantially reduced three days after MCAO, whereas the cell count of circulating tdTomato-labeled PI16^+^ FAPs was prominently increased (Supplementary Fig. S8e, f).

To further confirm the presence of cFAPs in stroke patients, a total of 22 age- and sex-matched healthy individuals and 38 stroke patients diagnosed within 3 days after symptom onset were included in this study. Stroke patients were categorized into two groups according to their NIHSS score: those with an NIHSS score ≤ 10 (mild group) and those with an NIHSS score > 10 (severe group). More detailed patient demographics are provided in Supplementary Table S1. Human FAPs were separated by flow cytometry as a subset of CD45^−^CD56^−^PDGFRα^+^CD15^+^ cells as previously described^29^. Compared with those in healthy controls, the number of circulating CD45^−^CD56^−^PDGFRα^+^CD15^+^ FAPs in severe stroke patients, but not mild stroke patients, increased rapidly during the acute phase of stroke (Fig. 2m, n). Furthermore, both the FAP count (*R* = 0.556, *P* < 0.0001) and frequency (*R* = 0.443, *P* = 0.000386) in peripheral blood were positively correlated with the NIHSS score (Fig. 2o). The relatively modest correlation strength may be due to the limited sample size and inherent inter-individual variability among the enrolled patients. These results indicate that stroke potentially mobilizes muscle-resident FAPs into circulation and that the increase in cFAPs could serve as a prognostic indicator of detrimental outcomes in stroke patients.

To elucidate the causal relationship between FAP loss and muscle deterioration, we crossed *PDGFRα-CreER* mice with *ROSA26-iDTR* mice followed by tamoxifen and diphtheria toxin administration (Supplementary Fig. S9a). Immunostaining revealed an approximately 70% reduction in PDGFRα intensity (Supplementary Fig. S9b). Consistent with the results of previous studies^15,19^, we found that ablation of FAPs in a homeostatic state led to rapid body weight loss and muscle atrophy (Supplementary Fig. S9c–f), highlighting the indispensable role of FAPs in muscle maintenance. Taken together, these results shed light on the previously unknown role of FAPs, whereby their migration from their niche into the circulation is a potential mechanism underlying muscle deterioration after stroke.

### Norepinephrine (NE) surge drives FAP loss and subsequent sarcopenia in MCAO mice

**Fig. 3:**
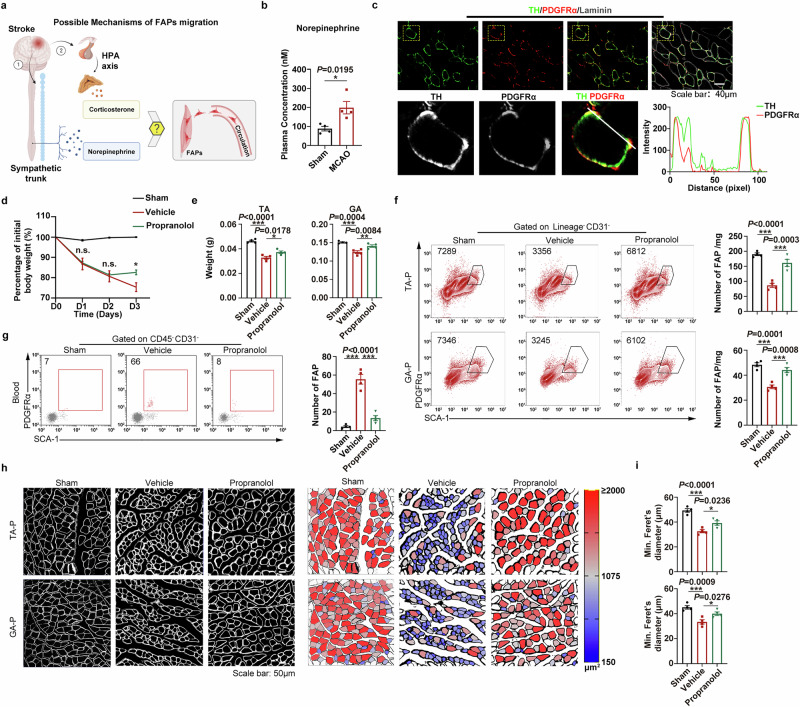
The β-blocker propranolol restores FAPs and muscle mass in MCAO mice. **a** Possible mechanisms of FAP mobilization. **b** The concentration of plasma NE was measured 3 days after MCAO. *n* = 4. **c** TH^+^ sympathetic fibers (green) innervated PDGFRα^+^ FAP (red) homeostasis. **d**, **e** Quantification of body weight (**d**) and absolute muscle weight (**e**) after 3 days of propranolol treatment in MCAO mice. *n* = 4. **f**, **g** FACS panels showing the number of PDGFRα^+^ SCA-1^+^ FAPs in paretic limb muscles (**f**) and peripheral blood (**g**). *n* = 4. **h** Histological assessment of TA and GA muscles from the paretic side of propranolol-treated mice and vehicle-treated control mice. *n* = 4. **i** Quantification of the myofiber minimal Feret’s diameter of the TA and GA muscles. *n* = 4.

Overactivation of the SNS and hypothalamus-pituitary-adrenal (HPA) axis has been widely observed in cerebrovascular patients and is widely recognized as the main cause of excessive catabolism and early weight loss after stroke^10,30,31^. Intriguingly, both catecholamine and glucocorticoid surges have been reported to promote stress fiber formation and stem cell mobilization^32,33^, suggesting their potential role in mediating FAP emigration. Therefore, we next explored the above-mentioned potential mechanisms of FAP migration and subsequent sarcopenia (Fig. 3a). To test whether excessive activation of the HPA axis is involved in stroke-related sarcopenia, we exposed experimental mice to the competitive glucocorticoid receptor (GR) antagonist mifepristone (10 mg/kg/d) for 3 days after MCAO. However, post-stroke mifepristone treatment failed to alleviate the loss of muscle-resident FAPs or skeletal muscle mass, suggesting that corticosterone signaling was not the major driver of stroke-induced FAP exhaustion (Supplementary Fig. S10).

We then investigated the sympathetic tone and detected increased NE release following MCAO surgery, as expected (Fig. 3b). Histological examination revealed that tyrosine hydroxylase (TH)^+^ sympathetic nerve fibers terminated in close proximity to FAPs (Fig. 3c; Supplementary Fig. S11a, b), suggesting the sympathetic innervation of FAP homeostasis. To determine whether sympathetic nerves indeed participate in FAP mobilization, we injected the β-adrenergic blocker propranolol (10 mg/kg/d) intraperitoneally for 3 consecutive days after MCAO to block adrenergic signaling. In general, comparable neurological deficit scores and infarct volumes were observed in vehicle- and propranolol-treated mice at 3 days after MCAO (Supplementary Fig. S12). Notably, compared with vehicle treatment, propranolol treatment restored both body weight and bilateral hindlimb muscle mass in control mice (Fig. 3d–e; Supplementary Fig. S13A). Furthermore, flow cytometric analysis revealed a significant increase in the number of muscle-resident FAPs and a reduction in the number of cFAPs (Fig. 3f, g; Supplementary Fig. S13b). Consistent with the reversal of FAP loss, systemic propranolol therapy markedly promoted the recovery of myofiber CSA in both paretic and non-paretic limb muscles (Fig. 3h, i; Supplementary Fig. S13c–e).

A previous study reported that the β-blocker carvedilol directly enhances muscle contractile performance through β-arrestin 1 signaling, a FAP-independent mechanism^34^. To investigate whether propranolol relieves stroke-related sarcopenia in a FAP-dependent manner, we intraperitoneally administered propranolol to FAP-depleted (*PDGFRα-CreER;Rosa26-iDTR*) mice for 3 consecutive days following MCAO (Supplementary Fig. S14a). Intriguingly, our data revealed that propranolol treatment had a minimal effect on body weight and muscle mass in FAP-deficient mice (Supplementary Fig. S14b, c). Additionally, no significant alterations in the CSA of myofibers or the expression of muscle atrophy genes were detected following propranolol treatment (Supplementary Fig. S14d–f). These findings suggest that the pro-hypertrophic effects of propranolol in stroke-related sarcopenia are dependent on FAPs. Overall, these data indicate that the systemic administration of propranolol following stroke, rather than mifepristone, may provide potent protection against the loss of muscle-interstitial FAPs as well as muscle deterioration.

In an attempt to determine whether the NE surge is sufficient to mobilize FAPs and cause sarcopenia, we implanted subcutaneous osmotic pumps to provide continuous delivery of either vehicle or NE for 3 days in C57BL/6 mice (Supplementary Fig. S15a). A dramatic increase in the plasma NE concentration was detected in NE-treated mice (Supplementary Fig. S15b), corroborating the efficacy of this strategy in mimicking stroke-induced catecholamine bursts. In the absence of stroke, sustained NE release elicited evident weight loss and skeletal muscle atrophy (Supplementary Fig. S15c–e). Furthermore, NE administration led to rapid induction of muscle FAP expression, concomitant with a reduction in myofiber CSA, which recapitulated muscle-related phenotypes in MCAO mice (Supplementary Fig. S15f–j). Collectively, our findings suggest that increased sympathetic tone and NE secretion are mainly responsible for post-stroke FAP depletion and muscle wasting.

### ADRB2 signaling mediates stroke-induced FAP exhaustion and sarcopenia

**Fig. 4:**
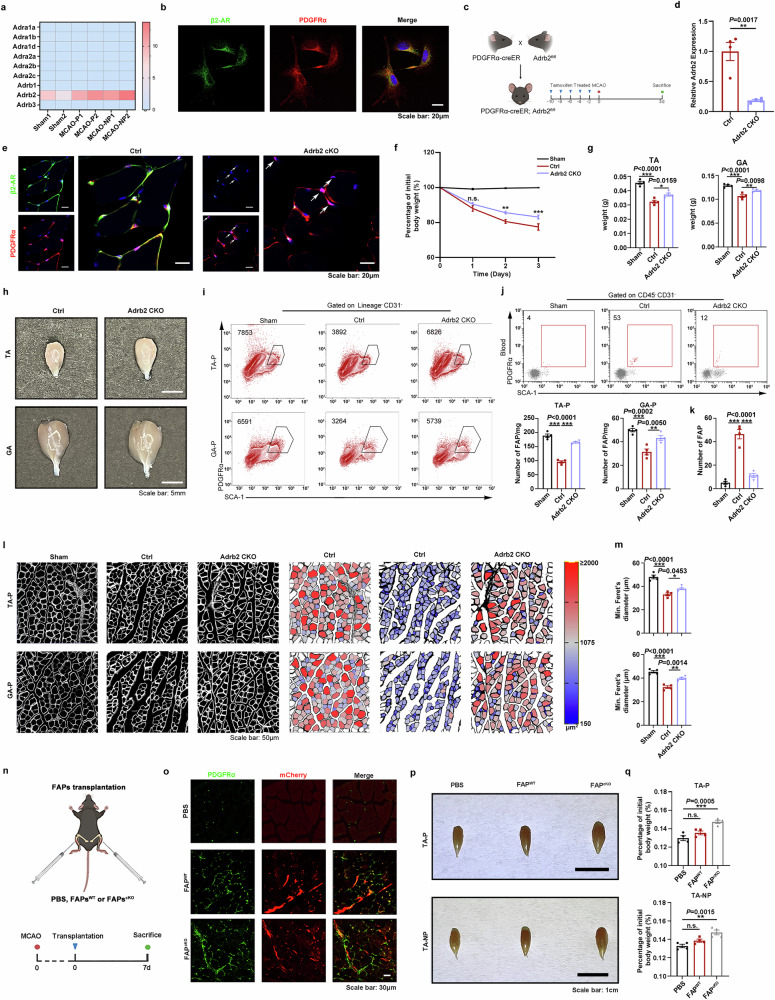
ADRB2 signaling mediates stroke-induced FAP exhaustion and sarcopenia. **a** Heatmap of the relative expression levels of genes encoding adrenergic receptors in isolated FAPs. **b** Immunofluorescence staining for the detection of ADRB2 (green) expression in PDGFRα^+^ (red) FAPs. **c** Experimental scheme for analyzing vehicle-treated control and *Adrb2* cKO mice on Day 3 after MCAO surgery. **d** The knockout efficiency was assessed by qRT-PCR in FAPs isolated from *Adrb2* cKO mice. *n* = 4. **e** Immunofluorescence staining for ADRB2 (green) and PDGFRα (red) in muscle sections from *Adrb2*-cKO mice to evaluate knockout efficiency. **f** *Adrb2* cKO blunted the body weight loss induced by MCAO. *n* = 4. **g** TA and GA muscle weights of vehicle-treated control and *Adrb2* cKO mice at 3 days after MCAO. *n* = 4. **h** Representative photographs of paretic TA and GA from control and *Adrb2* cKO mice at 3 days after MCAO. **i** FACS profiles for assessing the number of PDGFRα^+^ SCA-1^+^ FAPs in the paretic limb muscles of control and *Adrb2* cKO mice at 3 days after MCAO surgery. *n* = 4. **j**, **k** The numbers of PDGFRα^+^ SCA-1^+^ FAPs in peripheral blood were quantified by FACS in control or *Adrb2*-cKO mice 3 days after MCAO. *n* = 4. **l**, **m** Measurement of myofiber size by laminin staining of muscle cross-sections (**l**). The graphs indicate that the paretic muscle Min. Feret’s diameter (**m**) markedly increased in *Adrb2* cKO mice compared with vehicle-treated control mice. *n* = 4. **n** Scheme of the transplantation of WT and *Adrb2*-cKO FAPs. **o** Representative images showing the successful transplantation of mCherry-labeled FAPs into TA muscle. **p** Representative photographs of paretic or non-paretic TA muscle from FAP-transplanted mice 7 days after stroke. **q** Assessment of paretic or non-paretic TA muscle weights at 7 days after FAP transplantation. *n* = 4.

To elucidate the downstream signaling cascade of SNS activation, we isolated FAPs using FACS and examined the expression profiles of genes encoding adrenergic receptors. Notably, compared with other cognate receptors, FAPs predominantly express the β2-adrenergic receptor (ADRB2) (Fig. 4a). Immunofluorescence co-staining reconfirmed the expression of ADRB2 in cultured PDGFRα^+^ FAPs in vitro (Fig. 4b). To verify whether NE-induced activation of ADRB2 signaling directly induced FAP migration, we depleted ADRB2 from FAPs using *PDGFRα-CreER; Adrb2 flox/flox* mice, hereafter referred to as *Adrb2* conditional knockout (cKO) mice (Fig. 4c). After 5 days of tamoxifen treatment, both the mRNA and protein expression levels of ADRB2 decreased in PDGFRα^+^ FAPs from *Adrb2* cKO mice (Fig. 4d, e). We subsequently performed MCAO surgery to induce sarcopenia in both tamoxifen-induced *Adrb2* cKO mice and their littermate counterparts. In contrast to those in control mice, the decreases in body weight and skeletal muscle mass were blunted in *Adrb2* cKO mice at 3 days after MCAO (Fig. 4f–h; Supplementary Fig. S16a, b). Moreover, the sarcopenia-related phenotypes, including FAP release and a reduction in myofiber CSA, were also rescued by tamoxifen administration (Fig. 4i–m; Supplementary Fig. S16c–f). These results show that the FAP-specific ablation of *Adrb2* could partially abrogate the negative effects of stroke on bilateral skeletal myofibers.

To further investigate the therapeutic potential of cell therapy, we transplanted 2 × 10^5^ freshly isolated FAPs from unperturbed WT and *PDGFRα-CreER;Adrb2 flox/flox* mice (hereafter referred to as WT FAPs and *Adrb2* cKO FAPs, respectively) into the bilateral hind limb muscle of MCAO mice immediately after reperfusion. The recipient mice were then administered tamoxifen intraperitoneally for 3 consecutive days to deplete *Adrb2* in transplanted PDGFRα^+^ FAPs (Fig. 4n, o). Notably, 7 days post-transplantation, compared with the PBS-treated MCAO mice, the MCAO mice transplanted with *Adrb2*-cKO FAPs exhibited considerable increases in myofiber CSA and muscle weight. However, WT FAP transplantation did not notably affect the sarcopenic phenotypes induced by stroke (Fig. 4p, q). These results reinforced the functional role of ADRB2 signaling in stroke-induced FAP loss and subsequent sarcopenia. In summary, these results indicate that the replenishment of *Adrb2*-deficient FAPs partially counteracted stroke-related sarcopenia. Taken together, these results highlight that NE signaling via ADRB2 is the principal initiator of stroke-induced FAP exhaustion and sarcopenia and that the replenishment of *Adrb2*-deficient FAPs partially counteracts stroke-related sarcopenia.

### NE primes FAPs for mobilization by activating pro-migratory signals and degrading extracellular matrix (ECM) components

**Fig. 5:**
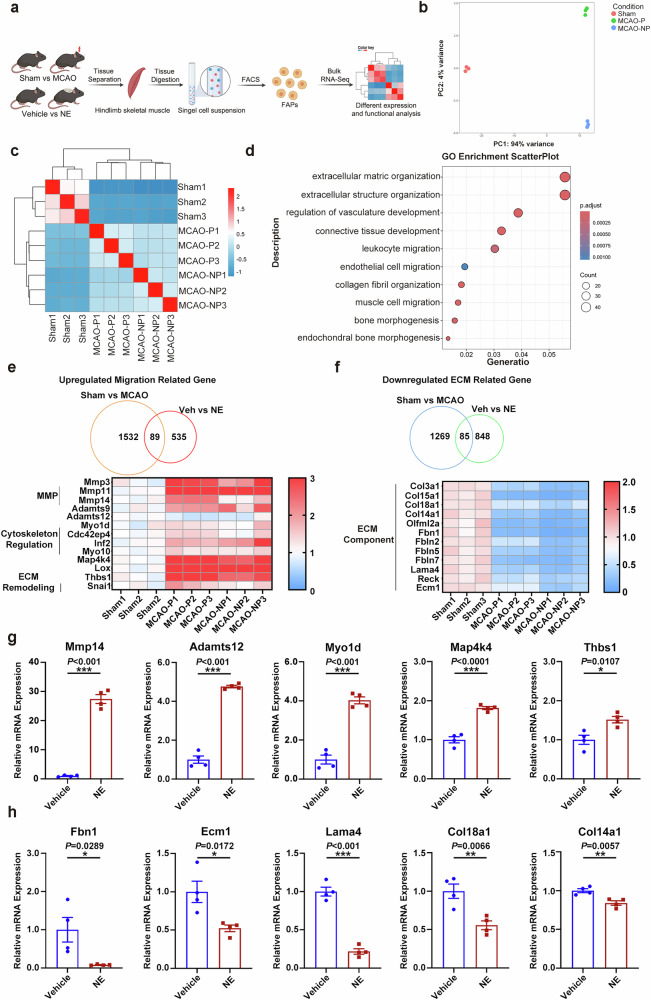
NE primes FAP for mobilization by activating pro-migratory signals and degrading ECM components. **a** Schematic diagram showing the workflow for bulk RNA-seq analysis of murine FAPs. **b** PCA plots showing a separation of FAPs isolated from the TA muscle of sham or MCAO mice. *n* = 3. **c** Sample clustering based on bulk RNA-seq data from the FAPs of sham-operated or MCAO mice. **d** GO enrichment analysis of genes that were differentially expressed between the FAPs from the MCAO mice and those from the sham-operated group. **e**, **f** Identification of signature migration- and ECM-related genes from each cluster of FAPs. **g**, **h** qRT-PCR analysis of upregulated migration genes (**g**) and downregulated ECM component genes (**h**) in cultured primary FAPs treated with NE. *n* = 4.

To elucidate the molecular mechanisms that elicit FAP mobilization, FAPs were freshly isolated by FACS from the muscles of paretic and non-paretic hindlimbs on the basis of the abovementioned expression profiles of cell surface markers. Afterward, we performed bulk RNA-seq analysis of FAPs by collecting RNA samples immediately after the isolation of FAPs from the hindlimb muscles of sham-operated mice and MCAO mice (Fig. 5a). Notably, principal component analysis (PCA) revealed a clustering tendency among the FAPs harvested from the MCAO-P and MCAO-NP groups, whereas the FAPs in the sham group formed another separate cluster, suggesting that MCAO induced the transcription of distinct FAPs (Fig. 5b). Moreover, Pearson’s correlation coefficient analysis (PCCA) revealed strong correlations among biological replicates of the MCAO-P and MCAO-NP groups but a weaker correlation between the sham and MCAO groups (Fig. 5c). These findings indicate that FAPs from the MCAO-P and MCAO-NP groups present similar gene expression signatures, whereas FAPs from sham-operated mice present a distinct RNA profile.

We subsequently conducted GO enrichment analysis to elucidate the major signaling pathways altered in FAPs after stroke. Among the DEGs, genes involved in the cell migration and ECM/structure organization GO term were extensively enriched, which could partially account for stroke-induced FAP migration (Fig. 5d). To validate whether the NE surge triggered similar alterations, we analyzed the changes in the transcription of purified FAPs from vehicle- and NE-treated C57BL/6 mice. Similarly, hierarchical clustering of DEGs and subsequent functional annotation analysis revealed significantly altered genes enriched in the ECM organization and cell motility sets (Supplementary Fig. S17). Next, we determined which pro-migratory genes were upregulated in the FAPs of both MCAO- and NE-treated mice. A certain portion of the increased migration-related transcripts in FAPs isolated from MCAO mice overlapped with those in NE-activated FAPs. In both instances, the expression of matrix metalloproteinases (MMPs), which are responsible for degrading ECM proteins and facilitating cell movement, was found to be elevated. Moreover, we also detected increased expression of genes involved in cytoskeletal rearrangement and cell migration, such as Myo1d and Map4k4 (Fig. 5e). In agreement with this augmented migratory phenotype, the expression of genes encoding ECM components was prominently reduced in FAPs after MCAO surgery or systemic NE administration. The expression of lysyl oxidase, which encodes a collagen crosslinking enzyme that facilitates ECM remodeling and metastasis, was also upregulated by sympathetic signaling (Fig. 5f). Some of these key changes were reconfirmed by qRT-PCR analysis of NE-treated FAPs in vitro (Fig. 5g, h). Thus, the stroke-induced NE surge primes FAPs for mobilization partially through the activation of pro-migratory signals and the degradation of ECM components.

To further assess the motility of FAPs under NE stimulation, we performed transwell migration assays and observed that the addition of NE (10 or 25 μM) to the lower chamber significantly increased the transmembrane migration of FAPs after 24 h of treatment (Supplementary Fig. S18a). Furthermore, the results of the scratch wound healing assays revealed increased mobility of FAPs upon NE stimulation (Supplementary Fig. S18b). Given the critical roles of MMPs and ADAMTS in FAP migration, we evaluated whether inhibiting these molecules could prevent FAP migration and the onset of sarcopenia. To test this hypothesis, we applied TAPI-2 (1 mg/kg), an inhibitor targeting MMPs and ADAMTS, to block their activity. As anticipated, inhibition of these ECM-degrading enzymes significantly reduced the migration of FAPs into the circulation and subsequently restored muscle mass (Supplementary Fig. S19).

### IGF-1 serves as the main effector exclusively derived from FAPs that preserves skeletal muscle homeostasis

**Fig. 6:**
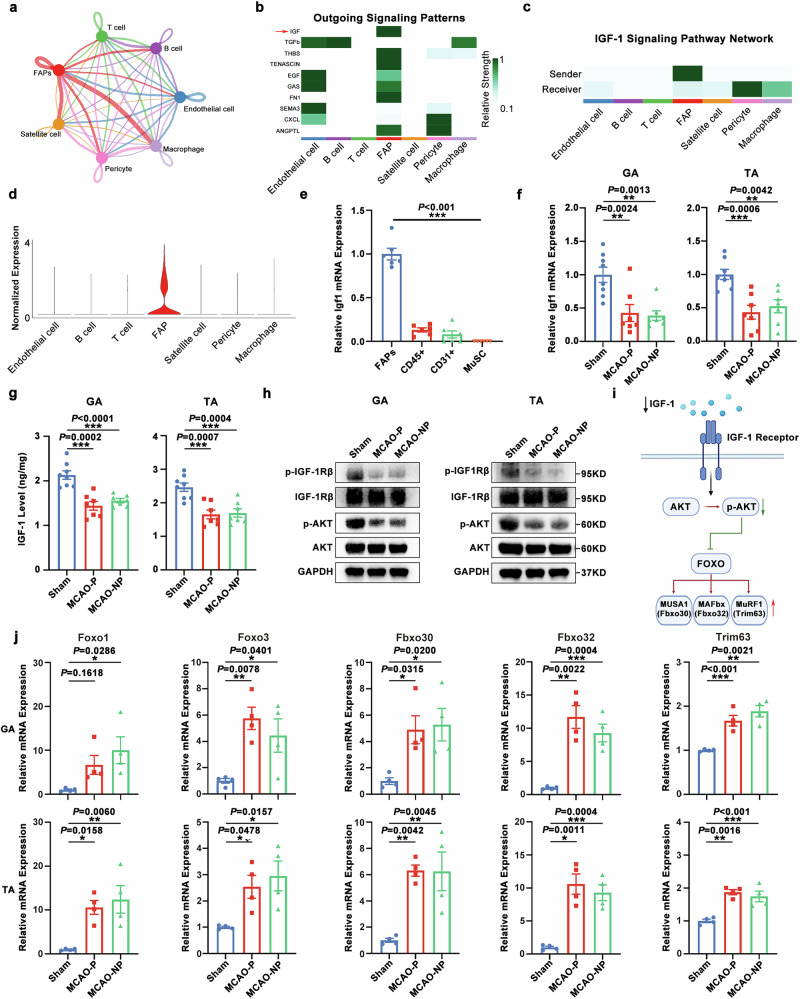
IGF-1 serves as the main effector derived from FAPs that preserves skeletal muscle homeostasis. **a** Circle plots showing inferred cell‒cell communication between any two cell subsets in skeletal muscle. **b** Outgoing signaling patterns of distinct cell clusters in skeletal muscle. **c** Relative contribution of each cell subset to the overall network of the IGF-1 signaling pathway in muscle. **d** The distribution of IGF-1 expression in skeletal muscle is shown as violin plots. **e** *Igf1* transcript abundance measured by qRT-PCR in different cell fractions, including FAPs, satellite cells, endothelial cells and hematopoietic cells. *n* = 6. **f**, **g** *Igf1* mRNA levels and concentrations in the bilateral limbs of MCAO mice were determined by qRT-PCR (**f**) and ELISA (**g**). Sham, *n* = 8; MCAO, *n* = 7. **h** Representative western blot images of phospho-IGF-1Rβ, total IGF-1Rβ, phospho-AKT, and total AKT expression in both the TA and GA muscles 3 days after stroke. **i** Schematic diagram illustrating the mechanism through which IGF-1 suppresses the expression of muscle atrophy genes, including Fbxo30, Fbxo32 and Trim63. **j** mRNA expression of muscle atrophy-related genes was measured by qRT-PCR in bilateral TA and GA muscle at 3 days after stroke. *n* = 4.

The advent of single-cell RNA-seq techniques enables exploration of the molecular characteristics of FAPs and elucidation of how FAPs mechanistically maintain myofiber size. We first processed published single-cell RNA-seq data from murine skeletal muscle^35^. By using CellChat to dissect cell‒cell communication^36^, we determined that FAPs serve as the principal signaling sender in the muscle microenvironment (Fig. 6a). Next, we investigated the key secreted factor responsible for the maintenance of FAP-dependent skeletal muscle homeostasis. A curated list of secreted proteins that are functionally pivotal for muscle maintenance was ranked by predicted interaction significance. CellChat analysis further quantified the differential expression of selected proteins within each cell cluster (Fig. 6b). Among the listed candidates, IGF-1 is among the most highly expressed trophic factors and is predominantly secreted by FAPs (Fig. 6c, d). Similar conclusions were drawn by analyzing a dataset^37^ from another study in which the RNA and chromatin accessibility of single myonuclei in adult skeletal muscles were sequenced (Supplementary Fig. S20). Despite detectable IGF-1 expression in other muscle niche components, such as MuSCs, immune cells, myofibers and tenocytes (Supplementary Fig. S21), qRT-PCR analysis revealed minimal transcript levels in satellite cells, CD45^+^ immune cells and CD31^+^ endothelial cells (Fig. 6e). At 3 days after MCAO, we detected a substantial reduction in IGF-1 expression in hindlimb muscle tissues (Fig. 6f, g). Therefore, we identified IGF-1 as a candidate molecule for FAP-mediated muscle homeostasis and focused on its downstream signaling.

As a potent muscle hypertrophy inducer, IGF-1 has been postulated to activate the IGF-1 receptor (IGF-1R), phosphorylate AKT and subsequently initiate anabolic signaling pathways^38,39^. Western blotting analysis revealed a pronounced reduction in the phosphorylation levels of IGF-1Rβ and AKT following MCAO but no prominent alterations in total IGF-1Rβ or AKT expression (Fig. 6h; Supplementary Fig. S22). Protein degradation mediated by E3 ubiquitin ligases, including muscle RING finger 1 (MuRF1, encoded by Trim63), muscle atrophy F-box (MAFbx, encoded by Fbxo32) and muscle ubiquitin ligase of the SCF complex in atrophy-1 (MUSA1, encoded by Fbxo30), are well-documented executors of skeletal muscle wasting. As expected, we detected a substantial increase in the FOXO-induced expression of atrophy-related ubiquitin ligases in the bilateral hindlimb muscle of MCAO mice (Fig. 6i, j). Collectively, these findings suggest that IGF-1 derived from FAPs may serve as a master regulator of muscle mass during the early stage of stroke. Specifically, loss of muscle-resident FAPs diminishes IGF-1 levels in the microenvironment, thereby suppressing AKT-FOXO signaling and leading to myofiber atrophy and dysfunction early after stroke onset.

**Fig. 7:**
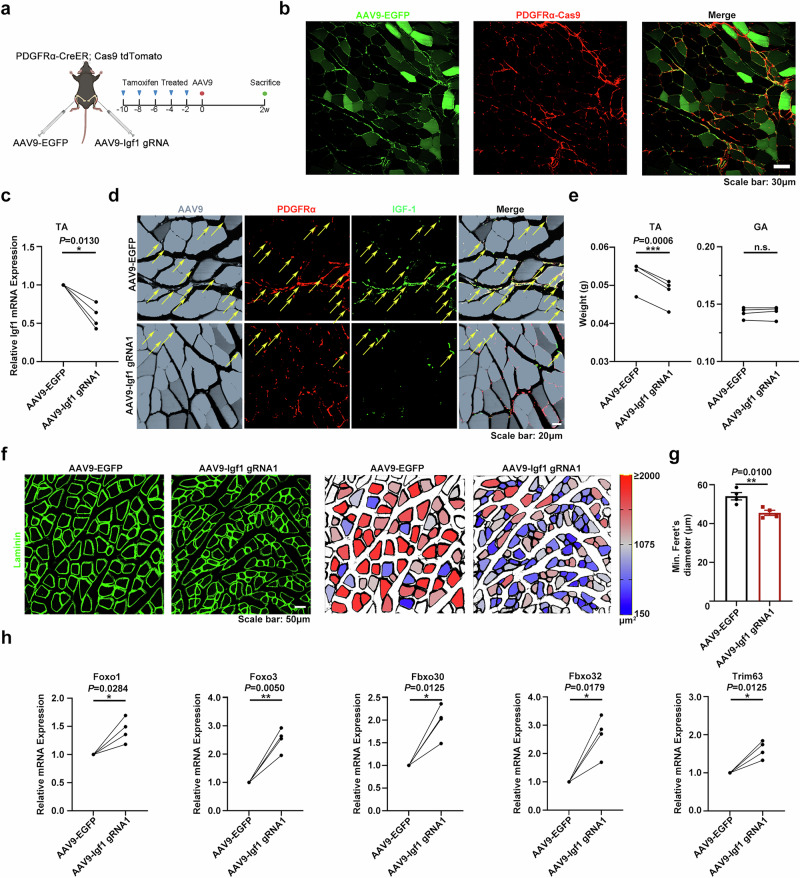
Deletion of *Igf1* in FAPs partially recapitulates the phenotypes of stroke-related sarcopenia. **a** Schematic of the experiment used to explore the influence of FAP-specific *Igf1* knockout on muscle homeostasis. **b** Representative images showing the infection efficiency of AAV9 in the FAPs of the TA muscle. **c**, **d** mRNA expression levels of *Igf1* (**c**) and protein abundance of IGF-1 (**d**) in AAV9-EGFP- or AAV9-*Igf1*-gRNA-injected TA muscle. *n* = 4. **e** The weight of the hind limb muscles at 2 weeks after AAV9 injection. *n* = 4. **f**, **g** Histological assessment of TA muscle from AAV9-EGFP- or AAV9-*Igf1*-gRNA1-injected *PDGFRα-CreER; Cas9-tdTomato* mice. Scale bar: 50 μm. *n* = 4. **h** mRNA expression of muscle atrophy-related genes in AAV9-EGFP- or AAV9-*Igf1*-gRNA1-injected TA muscle was measured by qRT-PCR. *n* = 4.

To obtain direct evidence of the function of FAP-secreted IGF-1 in vivo, we generated a FAP-specific *Igf1* cKO model using *PDGFRα-CreER; Rosa26-CAG-LSL-Cas9-tdTomato* mice (hereafter referred to as PDGFRα-Cas9 mice) and an AAV9-mediated guide RNA (gRNA) delivery system. PDGFRα-Cas9 mice were intraperitoneally administered tamoxifen (40 mg/kg) 5 times (every other day) and subsequently injected intramuscularly with AAV9-gRNA targeting *Igf1* in TA muscle (Fig. 7a). Two weeks after the intramuscular injection of AAV9-gRNA targeting *Igf1*, we assessed the transduction efficiency of AAV9 into FAPs on the basis of the co-expression of EGFP in the AAV9 vector backbone and tdTomato in the TA muscle of PDGFRα-Cas9 mice (Fig. 7b). Since gRNA-1 targeting *Igf1* exon 3 showed higher editing efficiency when compared to gRNA-2, gRNA-1 was chosen for the following experiments (Fig. 7c, d; Supplementary Fig. S23). Morphometric analysis revealed that the FAP-specific deletion of *Igf1* phenocopied stroke-induced muscle defects, including muscle weight loss and a reduction in CSA (Fig. 7e–g). Additionally, the mRNA levels of atrophy-related genes were markedly lower in the TA muscle of mice that received AAV9-*Igf1* gRNA-EGFP than in that of mice that received AAV9-EGFP control (Fig. 7h). Notably, the stroke-induced increase in IGF-1 expression in skeletal muscle could also be partially reversed by propranolol treatment and the conditional knockout of *Ardb2* (Supplementary Fig. S24), highlighting the essential role of FAP-derived IGF-1 in these two models.

Given the central role of IGF-1 in stroke-related sarcopenia, we next explored whether exogenous administration of IGF-1 could counteract muscle atrophy caused by FAP progression following MCAO. The experimental mice were subjected to intraperitoneal injection of recombinant IGF-1 protein (1 mg/kg) at 4 h after reperfusion, which was repeated daily for 3 days. Notably, compared with vehicle-treated mice, post-stroke mice treated with IGF-1 exhibited increased body weight and muscle size (Supplementary Fig. S25a–c). Similarly, the upregulation of Foxo and downstream E3 ubiquitin ligases, including Trim63 and Fbxo30, was attenuated by IGF-1 treatment (Supplementary Fig. S25d). Taken together, these results suggest that FAPs modulate muscle homeostasis primarily through the secretion of IGF-1. Restoring FAP-derived IGF-1 levels in the muscle niche partially enhances muscle mass and function after stroke.

## Discussion

Compared with other populations, such as cancer patients and elderly individuals, few studies have focused on the occurrence of sarcopenia in stroke patients, which can make it a blind spot of guideline recommendations. Muscle wasting not only compromises physical rehabilitation and long-term clinical outcomes but also predisposes stroke patients to future metabolic disorders^40^. The deleterious consequences of muscle wasting and the lack of effective strategies to stop its progression highlight the need to elucidate the cellular and molecular mechanisms involved in stroke-related sarcopenia. In the present study, we provide experimental evidence supporting the idea that acute stroke mobilizes FAPs to egress from the skeletal muscle niche, leading to a precipitous loss of muscle-resident FAPs and rapid onset of sarcopenia. Mechanistically, we report that increased β2-adrenergic signaling primes FAPs for mobilization by activating pro-migratory signals and degrading ECM components. Ultimately, IGF-1 was identified as the pivotal effector predominantly secreted by FAPs, which at least partially explains the supportive role of FAPs in the muscle niche.

FAPs are muscle-specific mesenchymal stromal cells that reside in the interstitial space between myofibers^41^. This perivascular location receives dense nerve fiber projections running along the blood vessels, which makes it an ideal position to respond first to local and systemic alterations^42^. Kaneshige et al. demonstrated that FAPs initially sense elevated local mechanical load and facilitate MuSC proliferation through paracrine THBS1 activity^43^. In response to acute muscle injury, FAPs convert inflammatory perturbations into cues that functionally promote immune infiltration^44^. Nevertheless, whether and how stress hormones regulate the cell fate of FAPs and contribute to stroke-induced sarcopenia remain unknown. In the present study, we showed that MCAO surgery, along with excessive activation of the SNS, promotes the migration of FAPs from the muscle niche into the circulation and culminates in muscle atrophy. Additionally, targeted disruption of ADRB2 signaling in FAPs restores muscle mass and hampers the progression of FAPs following stroke. In light of these observations, we hypothesize that NE-ADRB2 signaling directly induces FAP emigration and muscle wasting, which highlights the prominent role of neuroendocrine-mediated FAP loss in the development of post-stroke sarcopenia.

Acute stroke, also known as cerebral accident, usually provokes early and robust neuroendocrine responses through overactivation of the SNS and the HPA axis^45^. This surge in stress hormones is thought to account for the emergence of multiple stroke-induced systemic comorbidities, including but not confined to, immunosuppression, an imbalance in energy/glucose metabolism and cardiac dysfunction^46–48^. Furthermore, catecholamines and corticosteroids have been linked to the activation of catabolic signaling pathways and the progression of debilitating critical illness myopathy^49^. Previous research has revealed that pretreatment with the GR antagonist mifepristone can counteract the increased expression of myostatin and muscle atrophy caused by burn injury^50^. However, our study revealed no beneficial effects of this drug on stroke-related sarcopenia. These findings may be due to differences in dosing time but more likely suggest that glucocorticoids are not the major contributors to muscle wasting in the experiments described here. Given that corticosterone promotes inflammation resolution as well as acute lipolysis^51,52^, we found that mifepristone significantly restored the spleen weight and that the adipose tissue weight tended to increase (data not shown). We propose that these effects may partially account for the variability and increasing trend in the body weight of mifepristone-treated MCAO mice. On the other hand, sympathetic overactivity with increased circulating and local NE is increasingly being considered a cause of unfavorable functional outcomes in stroke patients^53^. In a large nonrandomized trial of 5212 stroke patients, Sykora and colleagues reported that β-blocker therapy protected patients from excessive sympathetic drive and reduced poststroke infection and mortality^54^. Here, we used β-blockers to determine whether sympathetic overactivation and innervation of FAP homeostasis could be responsible for poststroke muscle atrophy. We report, for the first time, that early anti-sympathetic treatment with propranolol could prevent the loss of muscle-resident FAPs as well as the development of stroke-related sarcopenia in MCAO mice. Moreover, a randomized prospective clinical trial has been registered and is being conducted to validate these findings clinically (www.chictr.org.cn, ChiCTR 2200057295), which may provide promising therapeutic options to treat stroke-related sarcopenia.

Emerging evidence has implicated tissue-resident MSCs as essential niche components that orchestrate tissue homeostasis and regeneration under both steady-state and pathological conditions^55^. Notably, recent studies have revealed that these tissue-resident MSCs function as initial sensors of environmental insults and relay pathological signals to parenchymal cells by providing mechanical support, immunomodulation, and trophic factors^56–58^. Adrenergic signals released from sympathetic nerve terminals directly act on surrounding MSCs to modulate immune responses and the progression of insulin resistance in gonadal adipose tissue^59^. These findings support the concept of neuro-mesenchymal unit and its role in maintaining tissue homeostasis^59^.

Neuroendocrine regulation of stem cell behaviors within the niche has been previously reported in bone marrow, intestine and other tissues^60^. Frenette et al. and other researchers have established that sympathetic outflow activates the migratory behaviors of murine hematopoietic stem/progenitor cells and leukocytes by locally altering the expression of chemokines and adhesion molecules^44,61^. Our results illustrate that this SNS-mediated cell trafficking is not restricted to the bone marrow but also occurs in other vital tissues, such as skeletal muscle. Using transcriptomic profiling, we revealed that NE conveys hormone relay to FAPs, which promotes cytoskeletal rearrangement and disrupts FAP retention in the skeletal muscle niche. These two mechanisms synergistically cooperate to increase the propensity of FAPs to dissociate from the muscle niche and mobilize these cells into circulation after MCAO. In stroke patients, an increase in the number of cFAPs is significantly correlated with stroke outcomes, as indicated by the NIHSS score. As a consequence, we propose that the increased number of cFAPs could serve as a predictor of unfavorable functional recovery in stroke patients. Targeting SNS-mediated FAP egress may open new avenues in the search for effective therapies to treat stroke-related sarcopenia. Future research is warranted to directly visualize FAP transendothelial migration through labeled cells and time-lapse imaging either ex vivo or in vivo.

In this study, we report that sympathetic overactivation elicits the migration of FAPs, a population of muscle-resident MSCs, from skeletal muscle into the circulation. The presence of circulating MSCs has previously been reported under conditions associated with heightened sympathetic tone, such as major skin injury and bone fractures, as detected by flow cytometry^62–64^. Recently, Sastourney et al. demonstrated that MSCs derived from subcutaneous adipose tissue (adipose stromal cells) are rapidly mobilized in response to muscle injury, contributing to an early increase in FAP abundance and facilitating muscle regeneration^65^. These findings align with and reinforce our observations, collectively supporting the concept that endogenous MSCs/FAPs can be mobilized from their resident niches upon pathological stimulation and trafficking within the circulation. To investigate whether the egress of MSCs is limited to skeletal muscle in stroke mice, we explored the changes in the number of MSCs in adipose tissue and bone marrow on Day 3 following MCAO. Notably, we found that stroke increased the number of MSCs in inguinal white adipose tissue (data not shown). These findings align with those of a previous report demonstrating that sympathetic activation via ADRB3 triggers a pronounced expansion of proliferating MSCs in WAT^66^. In the bone marrow, however, both ADRB2 and ADRB3 have been reported to regulate the differentiation fate of MSCs^67,68^, whereas propranolol treatment significantly promoted osteogenesis and inhibited adipogenesis of MSCs^69^. In MCAO mice, we detected a slight decreasing trend in the number of MSCs that did not significantly differ from that in sham-operated controls (data not shown). We therefore propose that the effects of NE and β-adrenergic receptor signaling on MSC biology are highly context dependent. The distinct outcomes, such as migration from skeletal muscle, expansion in adipose tissue, and differentiation alteration in the bone marrow, likely result from the activation of different predominant receptors (e.g., β₂AR vs β₃AR) and downstream pathways in various tissues and under specific pathological conditions. This tissue specificity highlights the sophisticated role of the SNS in orchestrating the responses of MSCs to maintain systemic homeostasis and provides a clear avenue for future investigations.

Moreover, recent studies have indicated that catecholamines can increase endothelial permeability by reorganizing the actin cytoskeleton and altering the membrane localization of key junctional proteins, thereby facilitating cell egress into the circulation^70^. To gain deeper mechanistic insight, future work should combine vascular permeability assays with direct visualization of FAP transmigration. Furthermore, investigations of other disorders characterized by both enhanced sympathetic signaling and sarcopenic phenotypes, such as pheochromocytoma and intensive unit acquired weakness^71,72^, are warranted to further validate the role of the NE-ADRB2 axis in FAP mobilization and sarcopenia progression.

Although the principal role of FAPs in sustaining muscle parenchyma has been illustrated by two recent FAP deletion studies, a thorough understanding of the mechanisms by which FAPs preserve muscle homeostasis is still lacking^15,19^. In a model of age-related sarcopenia, Uezumi et al. reported that a reduction in FAP-specific BMP3B expression was a causal factor for neuromuscular junction abnormalities and muscle atrophy^15^. Other researchers report that aging decreases the abundance of FAPs and induces the loss of the matricellular protein WISP1, which consequently perturbs the myogenic support of FAPs to MuSCs^73^. However, alterations in the behavior and secretome of FAPs during stroke-induced sarcopenia remain to be elucidated. Here, using an experimental stroke model with SNS hyperactivation, we observed that stroke induces the loss of muscle-resident FAPs. CellChat analysis of single-cell RNA-seq data further revealed that IGF-1 is specifically expressed by FAPs in skeletal muscle and potentially ensures muscle integrity through a paracrine mechanism. In line with previous findings, we observed that the compromised expression of IGF-1 activates FOXOs and increases the transcription of downstream pro-atrophic genes after stroke^74^. In addition, exogenous systemic administration of IGF-1 compromised LC3A/B-p62-mediated autophagocytosis in skeletal muscle tissue, which is well documented as downstream of FOXOs^75^, thereby preserving muscle mass.

However, a recent study by Luo et al. revealed that the deletion of *Igf1* in FAPs increases the senescence of FAPs and impairs differentiation but does not change skeletal muscle mass^76^. We hypothesize that the contradiction between these studies can be partially explained by key differences in genetic engineering and the body adaptive responses of the body. In the complete, constitutive knockout model used by Luo et al., the *Igf1* gene is absent from FAPs starting from the embryonic stage, which allows for developmental compensation. In contrast, our study utilized an inducible, conditional knockout model in adult mice, leading to partial loss of IGF-1. This acute deletion leaves no time for systemic compensation, thereby revealing the essential, non-redundant role of FAP-derived IGF-1 in post-natal muscle maintenance. Moreover, our findings are consistent with another recent work^77^, which also demonstrated that partial loss of IGF-1 expression, specifically in FAPs, leads to muscle atrophy. Clinically, several studies have demonstrated that lower IGF-1 levels are associated with higher mortality, poorer functional outcomes, and more comorbidities after ischemic stroke^78–80^. Additionally, a cross-sectional study involving chronic stroke patients revealed that reduced circulating IGF-1 is correlated with muscle atrophy and weakness^81^. However, as that study had a relatively small sample size (*n* = 14) and assessed IGF-1 and muscle function at only a single time point, further research is warranted to clarify the association between IGF-1 and post-stroke sarcopenia.

In conclusion, our results indicate that overactive SNS-induced FAP mobilization is the central cellular determinant of sarcopenia progression following stroke. Future studies are warranted to explore whether this sympathetic innervation of mesenchymal progenitors is conserved in other tissues, which represents a potential intervention target in critical illness settings. Further investigation of the detailed molecular mechanisms responsible for FAP migration and potential therapeutic approaches to preserve the ECM, such as collagen, may facilitate the sequestration of FAPs within the skeletal niche and alleviate muscle wasting. Moreover, we elucidated the pivotal role of FAP-secreted IGF-1 in suppressing catabolic signaling and maintaining skeletal muscle architecture in response to SNS overactivation. In support of this notion, early anti-sympathetic treatment with β-adrenergic blocking agents is a promising therapeutic strategy to counteract stroke-related sarcopenia.

## Materials and methods

### Human samples

Peripheral blood samples were collected from 22 healthy donors, 18 mild stroke patients and 20 severe stroke patients at the Third Affiliated Hospital of Sun Yat-sen University from February 2021 to December 2023 3–7 days. After stroke onset. All the participants provided informed consent. The NHISS score was evaluated on the day of admission and the day of blood collection. Clinical characteristics, including age, sex, risk factors and medications, were recorded. The demographic characteristics of stroke patients and healthy individuals are summarized in Supplementary Table S1. This clinical study was approved by the Ethics Committee of the Third Affiliated Hospital of Sun Yat-sen University ([2021]02-293-01) and registered with the Chinese Clinical Trial Registry (ChiCTR 2200057295).

### Animals

Male C57BL/6 mice aged 8‒10 weeks, ADRB2-flox mice (Stock# T052308), Pi16-CreERT2 mice (Stock# T057690) and Rosa26-CAG-LSL-Cas9-tdTomato mice (Stock# T002249) were purchased from GemPharmatech Co. PDGFRα-CreER mice were kindly provided by Professor Bo O. Zhou from the Chinese Academy of Sciences. Ai9 mice (Stock# 007909) and Rosa26-iDTR mice (Stock# 007900) were purchased from the Jackson Laboratory. All the animals were housed in individually ventilated cages under specific-pathogen-free conditions in the Laboratory Animal Center of Sun Yat-sen University. All experimental procedures were performed in accordance with protocols approved by the Ethics Committee of Sun Yat-sen University (#2021000777).

### Experimental model

To generate the MCAO model, in brief, the mice were anesthetized with 1.5% isoflurane and randomly divided into the sham and MCAO groups. All surgical instruments were sterilized before the operation. As previously described^82^, a 10-mm vertical incision was made on the midline of the neck to expose and isolate the right carotid artery, external carotid artery (ECA), and internal carotid artery (ICA). A silicone-coated suture was introduced into the ECA and then through the ICA to block the blood flow of the MCA. For reperfusion, the suture was withdrawn after 60 min, and the incision was sutured carefully. The sham group underwent the same anesthesia and surgical procedures but without artery occlusion. During surgery, a heating pad was used to maintain the core temperature of all the experimental mice at approximately 37 °C. The neurological deficit score was measured at 3 h after reperfusion and every 24 h for the following 3 days. A grading scale of 0–4 was used to assess neurological deficit after MCAO: 0, no obvious deficit; 1, weakness in the ipsilateral forelimb; 2, circulation to the ipsilateral side; 3, partial paralysis on the ipsilateral side; and 4, no spontaneous motor activity^83^. Humane endpoints were strictly enforced: any mouse exhibiting weight loss exceeding 30% or a neurological deficit score above 3 was promptly euthanized.

For the acute muscle injury model, an incision was made on the skin to fully expose the TA muscle after anesthesia. The TA muscle was then frozen with a liquid nitrogen-cooled metallic probe for 15 s to induce freezing injury as previously described^44^.

### Body weight measurement

Mouse body weights were measured with an electronic digital scale at the same time of day to exclude the influence of drinking and feeding. All measurements were normalized to the initial body weight recorded before the MCAO surgery.

### Drug administration

For tamoxifen treatment, the mice received a daily 40 mg/kg intraperitoneal injection of tamoxifen (T5648; Sigma‒Aldrich) diluted in corn oil for 5 consecutive days. For local delivery of tamoxifen metabolites to skeletal muscle tissue, endoxifen hydrochloride hydrate (Edx, E8284; Sigma‒Aldrich) was dissolved in methoxypolythylene glycol 350 (M6768-250G; Sigma) and mixed with melted polycaprolactone (PCL; 440752; Sigma‒Aldrich) at a 1:3 g/g ratio, as previously described^19^. After being thoroughly blended, the mixture was rapidly poured into an appropriate plastic mold to obtain a 1–2 mm thick sheet-like layer, which naturally solidified at room temperature. The sheets were carefully separated and weighed to ensure that each bar contained approximately 125 μg of Edx. The bars were then implanted subcutaneously above the TA with great care to avoid muscle damage and the incision was sutured after transplantation.

Propranolol hydrochloride (PHR1308; Sigma‒Aldrich) was dissolved in sterile saline, while mifepristone (M8036-500MG; Sigma) was dissolved in absolute ethanol and diluted with sesame oil (1:10) before use. Propranolol (10 mg/kg) or mifepristone (10 mg/kg) was intraperitoneally injected into the experimental mice 2 h before MCAO and at 6 h, 24 h, 48 h, and 72 h after MCAO. The mice in the vehicle group received an equivalent volume of sterile saline/sesame oil as a control.

For NE treatment, NE (N814761; Macklin) was dissolved in 0.9% sterile saline supplemented with 0.1% ascorbic acid to a final concentration of 10 mg/mL. NE solution was then loaded with mini-osmotic pumps (1003D, ALZET) at a release rate of 1 µL/h and implanted subcutaneously on the backs of the mice for 3 days. Identical pumps that dispensed 0.1% ascorbic acid/saline solution were implanted into the control group of mice.

For TAPI-2 treatment, TAPI-2 (HY-100211, MCE) was dissolved in PBS and intraperitoneally injected into experimental mice 2 h before MCAO and at 6 h, 24 h, 48 h, and 72 h after MCAO (1 mg/kg). The mice in the vehicle group received an equivalent volume of PBS.

### AAV9-mediated muscle-resident FAP labeling and IGF-1 ablation

Eight-week-old male *PDGFRα-CreER*;*Rosa26-CAG-LSL-Cas9-tdTomato* mice were treated with tamoxifen for 5 consecutive days before AAV9 administration. In brief, 40 μL of AAV9-gRNA (*Igf1*)-EGFP (5 × 10^10^ total vector genomes) was intramuscularly injected into the TA muscle of the left limb, and the same dose of AAV9-EGFP was injected into the right side as a control. Two weeks after AAV9 delivery, the bilateral TA muscles were harvested for further analyses. The gRNA-1 sequence was CGCTGGGCACGGATAGAGC, and the gRNA-2 sequence was GTCTGAGGTGCCCTCCGAAT.

### Flow cytometry

Individual skeletal muscle (GA and TA) and subcutaneous adipose tissue were isolated from mice at the indicated timepoints and placed in ice-cold PBS. Isolated muscle and adipose tissues were cut into small pieces of 2–4 mm^3^ and transferred to HBSS supplemented with calcium and magnesium, 0.1% BSA and 1 mg/mL collagenase II (17101015; Thermo Fisher) or 2 mg/ml collagenase I (17100017; Thermo Fisher) at 37 °C for 50 min with gentle agitation. Afterward, the digested tissue samples were passed through 40-μm filters to obtain single-cell suspensions. Peripheral blood samples were collected from the inferior vena cava of each mouse after anesthesia. EDTA solution (5 mM) was applied to prevent blood coagulation. Bone marrow samples were collected from the tibia cavities of experimental mice by flushing with a 25-gauge needle. Red blood cells (RBCs) in peripheral blood and bone marrow samples were removed with RBC lysis buffer (Bio-Gems) according to the manufacturer’s instructions. Single cells from skeletal muscle and peripheral blood were stained with fluorescent-dye-conjugated antibodies, including SCA-1-BV510 (1:100, 565507, BD), PDGFRα-APC (1:100, 562777, BD), CD31-PE-FITC (1:100, 558738, BD), CD45-PE-Cy5.5 (1:100, 15-0451-82, Thermo Fisher), CD31-PE-Cy7 (1:100, 25-0311-82, Thermo Fisher), Lineage Cocktail-FITC (1:100, 22-7770-72, Thermo Fisher) and Integrin α7-PE (1:100, AbLab).

For human peripheral blood, 1 mL of blood was collected from each healthy control or patient in anticoagulant tubes. RBCs were removed using RBC lysis buffer (11814389001; Roche). Single-cell suspensions were collected and subjected to further staining with CD45-PE-Cy7 (1:50; 557748; BD), PDGFRα-PE (1:50; 556002; BD), CD56-APC (1:50; 362504; BioLegend) and CD15-APC (1:50; 323040; BioLegend).

Cell sorting was performed using a fluorescence-activated cell sorter from BD Influx. Flow cytometry analysis was conducted with a CytoFLEX flow cytometer (Beckman Coulter), and the results were assessed with CytExpert software.

### Quantitative analyses of hormones and cytokines

Plasma NE levels were determined by KINGMED Diagnostics (Guangzhou), an independent clinical laboratory. Serum corticosterone levels were measured using an ELISA kit (K014-H1; Arbor Assays) following the manufacturer’s instructions.

To determine tissue IGF-1 levels, 20 mg muscle samples were isolated from the TA or GA, rinsed in ice-cold saline and homogenized with 1 mL of PBS. The homogenates were then centrifuged at 10,000 rpm for 10 min. The supernatant was collected, and the IGF-1 levels were detected by an ELISA kit (EMI1001-1; AssayPro) as recommended by the manufacturer.

### Protein extraction and western blotting

Protein was extracted from TAs and GAs using the Minute^TM^ Total Protein Extraction Kit for Muscles (SA-06-MS, Invent). The protein concentration was quantified by a BCA protein assay kit (Thermo Fisher Scientific). Once loaded and separated by SDS-PAGE, protein samples were transferred to 0.45-μm pore-sized polyvinylidenedifluoride (PVDF) membranes (Millipore) and blocked with 5% BSA in 1× TBST buffer. Next, we incubated the membranes with appropriate dilutions of primary antibodies, including anti-IGF-1 receptor β (1:1000; 9750S; Cell Signaling Technology), anti-phospho-IGF-1 receptor β (1:1000; 3021S; Cell Signaling Technology), anti-AKT (1:1000; 4691S; Cell Signaling Technology), anti-phospho-AKT (1:500; 4060S; Cell Signaling Technology), and anti-GAPDH (1:1000; 2118S; Cell Signaling Technology). Immunoreactivity was semiquantitatively measured by a ChemiDoc Imaging System (Bio-Rad).

### RNA isolation, reverse transcription, and qRT-PCR

CD45^+^ cells, CD31^+^ cells, FAPs and MuSCs were isolated from skeletal muscle using FACS. Total RNA from isolated cells or skeletal muscle tissues was extracted using an RNeasy Mini Kit (QIAGEN) following the manufacturer’s instructions. The extracted RNA samples were quantified with a Nanodrop 1000 spectrophotometer and reverse transcribed using a RevertAid First Strand cDNA Synthesis Kit (Thermo Fisher Scientific). Harvested cDNA samples were subsequently used as templates for qRT-PCR, which was performed in duplicate using SYBR Green qRT-PCR Super Mix (Roche) and detected with a Light Cycler 480 detection system (Roche). The primers used for the qRT-PCR analysis are listed in Supplementary Table S2.

### Low-input RNA-seq of cFAPs

Peripheral blood samples (~20 mL) were collected from 20 male MCAO C57BL/6 mice after anesthesia and anticoagulated with 5 mM EDTA solution. RBCs were then eliminated by RBC lysis buffer (Bio-Gems) according to the manufacturer’s instructions. The resulting single cells were subjected to staining with fluorescent-dye-conjugated antibodies, including those against SCA-1-BV510 (1:100; 565507; BD), CD31-PE-FITC (1:100; 558738; BD), PDGFRα-APC (1:100; 562777; BD) and CD45-PE-Cy5.5 (1:100; 15-0451-82; Thermo Fisher), for 30 min on ice. Cell sorting was conducted utilizing a BD Influx flow cytometer, and CD45^−^/CD31^−^/SCA-1^+^/PDGFRα^+^ cells were identified as cFAPs.

All the collected cFAPs were subjected to subsequent RNA extraction and cDNA synthesis procedures. Approximately 1500 FAPs were lysed to release all the RNA. cDNA was subsequently synthesized by using a SMARTer Ultra LowRNA Kit (Clontech Laboratories). In brief, mRNA was reverse transcribed into cDNA with SMARTScribe Reverse Transcriptase (Clontech Laboratories), and long-distance PCR was performed for 20 cycles for each sample. cDNA was further purified by SPRI AMPure Beads. Amplified and purified cDNA is digested with RsaI to remove the SMART adapter prior to sequencing. The quality of the cDNA was verified by DNA gel electrophoresis. The TruePrep DNA Library Prep Kit V2 for Illumina (TD503, Vazyme) was used to index 1 ng of cDNA from each sample according to the manufacturer’s instructions. Indexed cDNA libraries were sequenced using the Illumina Nova Xplus platform, yielding 150-bp paired-end reads. FastQC (version 0.11.2) was used to evaluate the quality of the sequenced data. The raw reads were trimmed by Trimmomatic (version 0.36). Clean reads were mapped to the mouse genome (grcm39) using STAR (version 2.7.11).

### Immunofluorescence

The mice were anesthetized and perfused with ice-cold saline followed by 4% paraformaldehyde (PFA) solution. Skeletal muscle tissues were carefully removed and fixed in 4% PFA overnight. The tissues were then transferred to 20% sucrose for dehydration and frozen with OCT (SAKURA). For further staining and analysis, the tissues were cut into 20-μm frozen cryosections using a microtome (CM1900, Leica). Both cryosections and cultured cells were fixed in 4% PFA for 20 min, permeabilized in 0.25% Triton X-100 PBS for 15 min, and blocked with 5% BSA/PBS for 1 h, after which they were incubated with the following antibodies: rabbit anti-Laminin (1:300; L9393; Sigma), goat anti-PDGFRα (1:100; AF1062; R&D), rabbit anti-PDGFRα (1:100; 3174S; Cell Signaling Technology), rabbit anti-tyrosine hydroxylase (1:100; ab152; Millipore), mouse anti-TUJ1 (1:100; 4466S; Cell Signaling Technology), rabbit anti-β2-AR (1:100; sc-81577; Santa Cruz), rabbit anti-p16INK4a (1:100; ab211543; Abcam), rat anti-F4/80 (1:100; ab6640; Abcam), rabbit anti-RIP3 (1:100; 95702S; Cell Signaling Technology), rabbit anti-MMP14 (1:100; ab51074; Abcam), and mouse anti-Scleraxis (1:50; sc-518082, Santa Cruz). A substitution of the primary antibody for isotype-specific immunoglobulins at the same protein concentration as the primary antibody was used as a negative control (1:100, 3900S; Cell Signaling Technology; 1:100, 5415S; Cell Signaling Technology). Nuclei were visualized by DAPI (Roche) staining for 10 min.

For apoptosis analysis, In Situ Cell Death Detection Fluorescein (Roche) was used for staining according to the manufacturer’s instructions. Immunofluorescence images were acquired using a DMi8 microscope (Leica), an LSM800 confocal microscope (Zeiss), and a Dragonfly high-speed confocal microscope (ANDOR, Oxford Instruments).

### Histological analyses

CSA measurements were performed on the whole TA muscle and individual myofibers after immunostaining for laminin. ImageJ software was used to measure the CSA of the whole TA muscle. To measure the CSA of myofibers, a range of 500–1000 representative myofibers from three separate cryosections were quantified per sample. The values are presented as the mean CSA and average percentage of the frequency distribution. To generate pseudocolor images of the CSA, laminin-stained images were processed with the ROI Color Coder macro in ImageJ.

To quantify the colocalization of PDGFRα^+^ FAPs and TH^+^ nerve fibers in skeletal muscle, we split the channels of the immunostaining images and applied a threshold to each channel. Colocalization was considered the presence of at least one overlapping pixel between two channels according to ImageJ software as previously described^58^.

The infarct volume of the brain sections was determined by 2,3,5-triphenyltetrazolium chloride (TTC; 2% dissolved in 0.9% saline) staining. In brief, freshly harvested brains were cut into 1 mm-thick coronal sections (1 mm thick), incubated with 2% TTC solution for 20 min at room temperature, and subsequently fixed with 4% PFA. The mean infarct area for each section was assessed and multiplied by the section thickness to calculate the infarct volume.

### Cell culture

Freshly isolated FAPs were cultured in high-glucose Dulbecco’s modified Eagle’s medium (DMEM) (Corning) supplemented with 20% fetal bovine serum (FBS; PAN Biotech) and 1% penicillin/streptomycin (Thermo Fisher). When the cultured FAPs reached 80%–90% confluence, they were continuously passaged using 0.25% trypsin-EDTA. FAPs at passages 3–8 were used for the in vitro experiments.

### Scratch assay

When the FAPs reached approximately 90% confluence, a straight scratch was made in the center of each well using a 200 μL sterile pipette tip held perpendicular to the culture plate. The cells were gently washed three times with PBS to remove detached cells. The medium was subsequently replaced with basal medium without fetal bovine serum to inhibit cell proliferation and supplemented with either vehicle, 10 µM, or 25 µM NE. Images of the scratch area were captured at 0 h and 24 h post-scratching using a microscope.

### Transwell assay

FAPs in the logarithmic growth phase were harvested and resuspended in serum-free basal medium, with the cell density adjusted to 1 × 10^6^ cells/mL. A 200 μL aliquot of the cell suspension was carefully added to the upper chamber of a 24-well Transwell plate. The lower chamber was filled with 600 μL of vehicle, 10 µM, or 25 µM NE. The plate was incubated at 37 °C with 5% CO₂ for 24 h. Following incubation, non-migrated cells on the upper surface of the membrane were mechanically removed using a cotton swab. The cells that migrated to the lower membrane surface were fixed with 4% paraformaldehyde for 15 min and stained with 0.1% crystal violet solution for 20 min at room temperature. After thorough washing with PBS, the membrane was air-dried and visualized under an inverted microscope.

### Statistical analysis

All the results are presented as the mean ± SEM of at least three independent experiments. The sample size of each experiment is indicated in the corresponding figure legend. Differences in the means among multiple groups were analyzed using one- or two-way analysis of variance (ANOVA). Student’s *t*-test was used for two-group comparisons. In all analyses, significance was defined as *P < 0.05, **P < 0.01, and ***P < 0.001.

## Supplementary information

Supplementary information
