# Supplementary material for: Sympathetic nervous system-mediated fibro-adipogenic progenitor mobilization drives stroke-related sarcopenia
Source: Cell Discov. 2026 Jul 7;12:49. doi: 10.1038/s41421-026-00899-0 (PMC13338466; doi:10.1038/s41421-026-00899-0)
Supplement: Supplementary file 1 — Supplementary information [file 41421_2026_899_MOESM1_ESM.pdf]

Sympathetic Nervous System-Mediated Fibro/Adipogenic Progenitor Mobilization Drives Stroke-Related Sarcopenia

Supplementary Table S1-2.

Supplementary Figures S1-25.

Supplementary Table S1. Demographics and baseline characteristics of the recruited patients.

	Control group (n=22)	Mild (n=18)	Severe (n=20)	P value (Mild vs. Severe)
Age, year, median (IQR)	65.9 (57-71)	69 (54-72)	61 (51-67)	0.1905
Male gender, N (%)	14 (63.6)	14 (77.8)	12 (60.0)	0.3067
**Risk factors, N (%)**				
Hypertension	-	13 (72.2)	8 (40.0)	0.0585
Diabetes	-	3 (16.7)	2 (10.0)	0.6525
Cardiac problems (e.g. atrial fibrillation)	-	1 (5.6)	4 (20.0)	0.3436
Smoking	-	5 (27.8)	3 (15.0)	0.4381
Dyslipidemia	-	0 (0)	0 (0)	>0.9999
Prior cerebrovascular events	-	1 (5.6)	2 (10)	0.6525
**Stroke subtype**				
Atherosclerosis	-	12 (66.7)	4 (20.0)	0.0076
Cardioembolic origin	-	0 (0)	0 (0)	>0.9999
Others	-	6 (33.3)	16 (80.0)	0.0076
Unknown	-	0	0	>0.9999
NIHSS score on admission, median (IQR)	-	3.5 (2-5)	40 (40-40)	<0.0001
**Medications**				
Aspirin/clopidogel	-	8 (44.4)	4 (20.0)	0.1643
Statin	-	11 (61.1)	4 (20.0)	0.0189
ACE inhibitor or ARB	-	2 (11.1)	3 (15.0)	>0.9999
Ca2+ channel blocker	-	4 (22.2)	4 (20.0)	>0.9999
Hypoglycemic agent	-	4 (22.2)	4 (20.0)	>0.9999
**Constitution of neutrophil at admission, median (quartiles)**				
FAPs count	314.5 (187-517)	484 (209-1084)	2837 (1112-4983)	0.0003
FAPs ratio (%)	0.0183 (0.0106-0.0374)	0.0204 (0.0117-0.0658)	0.0632 (0.0295-0.1525)	0.0764

Abbreviations: IQR = interquartile range; NIHSS = NIH Stroke Scale

Supplementary Table S2. Primer list of qPCR analysis.

	Forward	Reverse
Col14a1	TTTGGCGGCTGCTTGTTTC	CGCTTTTGTTGCAGTGTTCTG
Col18a1	GTGCCCATCGTCAACCTGAA	GACATCTCTGCCGTCAAAAGAA
Ecm1	GGGACCGTATCCAGAGCAG	GCTGGTCTGAAGCCTTGAAG
Fbn1	GGACGCCAATTTGGAGGCT	CTTTCAGCGCATCGTGTCCT
Foxo1	CCCAGGCCGGAGTTTAACC	GTTGCTCATAAAGTCGGTGCT
Foxo3	CTGGGGGAACCTGTCCTATG	TCATTCTGAACGCGCATGAAG
Gapdh	TCAAGAAGGTGGTGAAGCAG	GTTGAAGTCGCAGGAGACAA
Lama4	ATGAGCTGCAAGGAAAACTATCC	CTGTTTCGTTGGCTTCACTGA
Adamts12	CACGGGAGACAAACTCAGCC	CTGGCAGGGACTTGATAAAATGT
Map4k4	CTGGCCGCCATCAAGGTTAT	AGCACCATAGTACGTGGCAAT
Fbxo32	CAGCTTCGTGAGCGACCTC	GGCAGTCGAGAAGTCCAGTC
Mmp14	CAGTATGGCTACCTACCTCCAG	GCCTTGCCTGTCACTTGTAAA
Fbxo30	TCGTGGAATGGTAATCTTGC	CCTCCCGTTTCTCTATCACG
Myo1d	CCCAGTTGCTAATGAGCTGAA	AGTGACAAAAATTGCTCGGTCTT
Thbs1	GGGGAGATAACGGTGTGTTTG	CGGGGATCAGGTTGGCATT
Trim63	GTGTGAGGTGCCTACTTGCTC	GCTCAGTCTTCTGTCCTTGGA
Igf1	CTGGACCAGAGACCCTTTGC	GGACGGGGACTTCTGAGTCTT

Supplementary Figures.

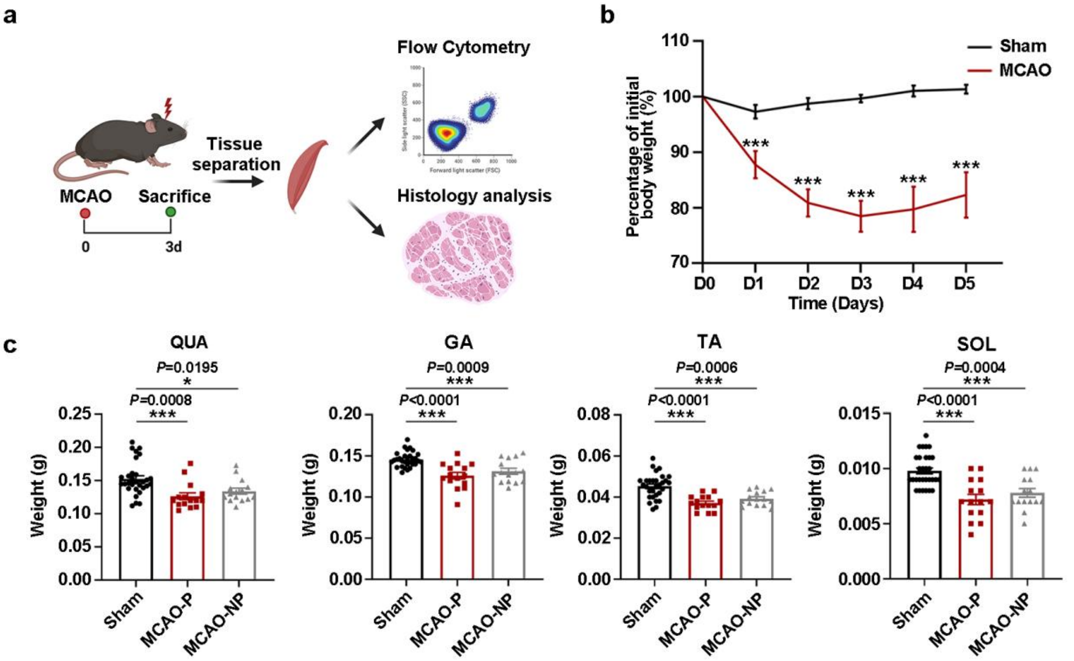

Supplementary Figure S1. MCAO mice exhibit early weight loss and muscle dysfunction after stroke.

(a) Schematic diagram of the MCAO procedure.

(b) Temporal dynamics of body weight change after stroke. n=15.

(c) Assessment of muscle weight at post-stroke day 3. (QUA. quadriceps, SOL. soleus, TA. tibialis anterior, GA. gastrocnemius). n=15.

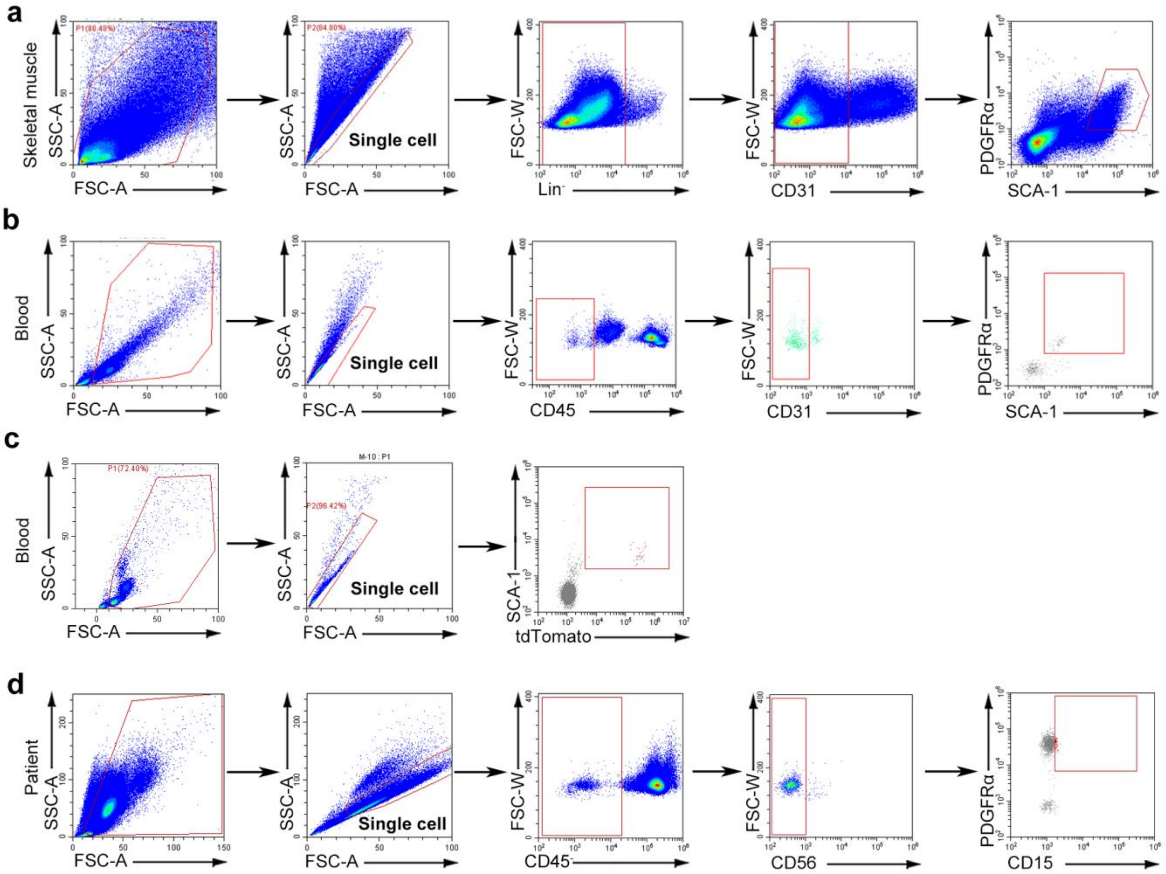

Supplementary Figure S2. Gating strategy of muscle FAPs (a), cFAPs (b), SCA-1$^+$ tdTomato$^+$ cells in blood (c) and CD45$^-$CD56$^-$CD15$^+$PDGFRα$^+$ in patients' blood (d).

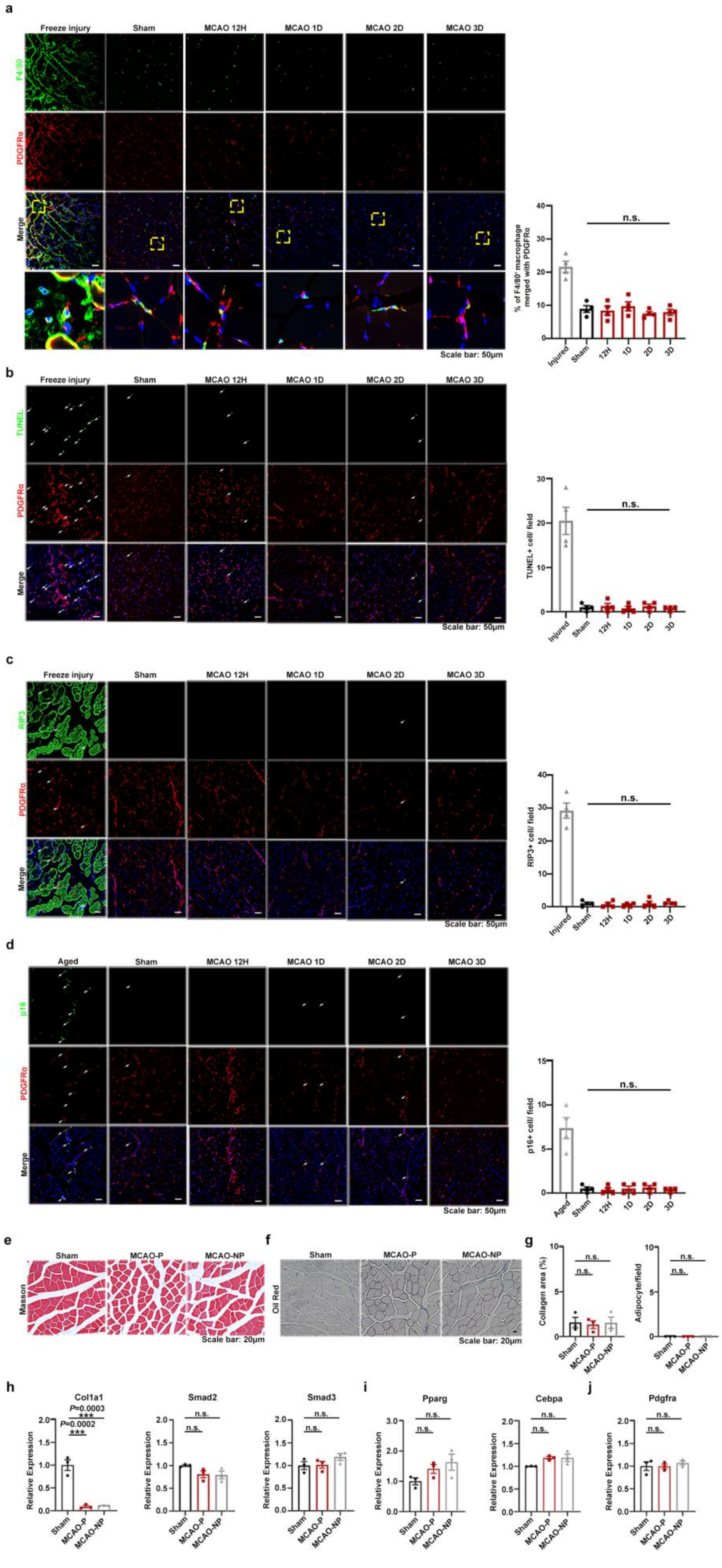
a
Freeze injury
Sham
MCAO 12H
MCAO 1D
MCAO 2D
MCAO 3D
F4/80
PDGFRα
Merge
Scale bar: 50μm
% of F4/80+ macrophage merged with PDGFRα
n.s.
Injured
Sham
12H
1D
2D
3D
b
TUNEL
PDGFRα
Merge
TUNEL+ cell/ field
c
RIP3
RIP3+ cell/ field
d
Aged
p16
p16+ cell/ field
e
Sham
MCAO-P
MCAO-NP
Masson
Scale bar: 20μm
f
Oil Red
g
Collagen area (%)
Adipocyte/field
h
Col1a1
P=0.0003
***
P=0.0002
***
Smad2
Smad3
Relative Expression
i
Pparg
Cebpa
j
Pdgfra

Supplementary Figure S3. Analysis of apoptosis, senescence, necrosis, differentiation and engulfment of FAPs at different time points following stroke.

(a) Engulfment of PDGFRα$^+$ FAPs (red) by F4/80$^+$ macrophage (green) in TA sections of MCAO mice 12 hours to 3 days after stroke. Scale bar: 50μm.

(b-d) Immunofluorescence staining and quantification of TUNEL (b), necrosis marker RIP3 (c) and senescence marker p16 (d), in TA sections of MCAO mice 12 hours to 3 days after stroke. Freeze-injured muscle sections were used as positive controls to detect cell apoptosis, necrosis, and phagocytosis, while 24-month-old age mice muscle sections were adopted as positive controls to determine cell senescence. Scale bar: 50μm. n=4.

(e) Masson's Trichrome staining in muscle sections from sham and stroke mice. Scale bar: 20μm.

(f) Oil Red staining in muscle sections from sham and stroke mice. Scale bar: 20μm.

(g) Quantification analysis of collagen area and adipocyte number in TA muscle sections. n=3.

(h-i) Relative expression of pro-fibrotic (h) and adipogenic (i) genes in isolated FAPs. n=3.

(j) Relative expression of PDGFRα in FAPs isolated from sham-operated and MCAO mice. n=3.

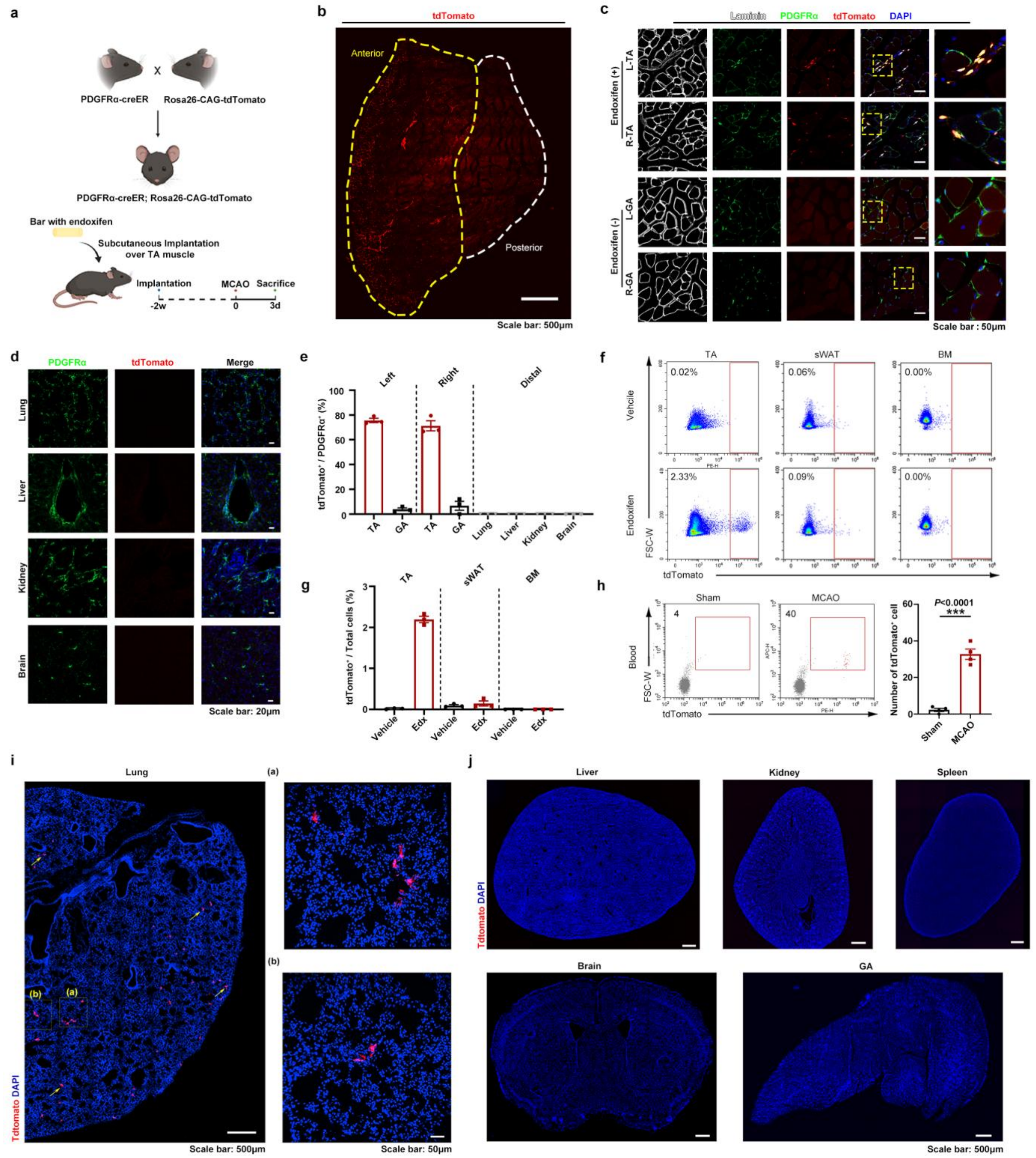

Supplementary Figure S4. Stroke-induced FAPs egression in *PDGFRα-CreER; Ai9* mice implanted with the Edx/PCL bar.

(a) Schematic diagram showing genetic-lineage-tracing strategy for PDGFRα-tdTomato$^+$ FAPs derived from the TA muscle.

(b) Confocal images taken from whole TA and GA cross sections illustrated the tissue efficiency and specificity of Cre-dependent tdTomato expression in PDGFRα$^+$ FAPs. Scale bar: 500 μm.

(c) Representative images showed that PDGFRα-tdTomato-labeled FAPs distributed in the muscle interstitium, suggesting a specific recombination in *PDGFRα-CreER;Ai9* mice. Scale bar: 50 μm.

(d-e) Representative images (d) and quantification (e) of Cre-mediated tdTomato expression in the lung, liver, kidney, and brain. Scale bar: 20 μm.

(f-g) Representative FACS panels (f) and quantification (g) of the percentage of tdTomato-labeled FAPs following Edx/PCL implantation in TA, subcutaneous white adipose tissue (sWAT) and bone marrow (BM). n=3.

(h) Representative FACS panels showed the number of PDGFRα-tdTomato-labeled FAPs derived from the TA muscle in the peripheral blood at 1 day after MCAO. n=4

(i) Representative fluorescence images demonstrating the tdTomato-labeled FAPs derived from TA distributed in the lung after stroke.

(j) Fluorescence images showing few tdTomato-labeled FAPs was distributed in the liver, kidney, spleen, brain, and GA muscle after stroke.

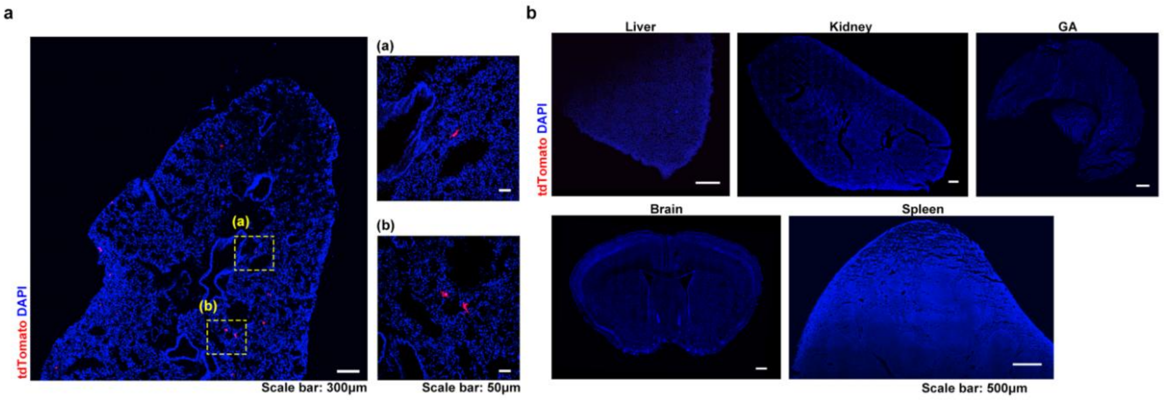

Supplementary Figure S5. Tracing the trajectory of TA-derived FAPs using *PDGFRα-CreER;* mice intramuscularly injected with AAV9-DIO-tdTomato.

(a) Fluorescence images demonstrating the distribution of tdTomato-labeled FAPs derived from TA in the lung after stroke. Scale bar: 300/50μm.

(b) Fluorescence images showing tdTomato-labeled FAPs were barely detected in the liver, kidney, spleen, brain, and GA muscle after stroke. Scale bar: 500μm.

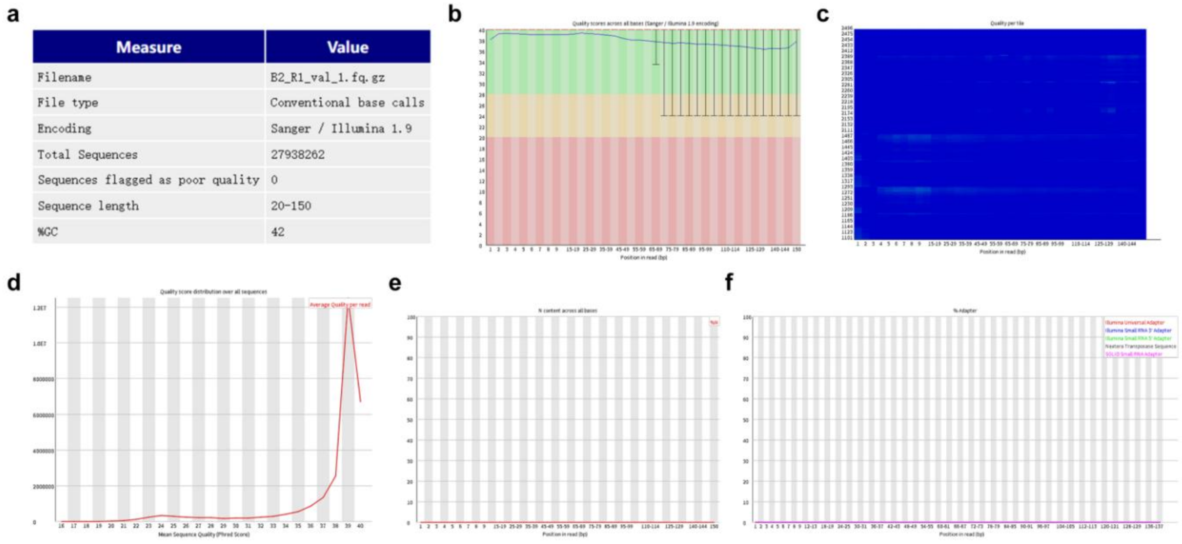

Supplementary Figure S6. Representative images respectively showing the basic statistics (a), per base sequence quality (b), per tile sequence quality (c), per sequence quality scores (d), per base N content (e) and adapter content (f) of low input RNA-seq.

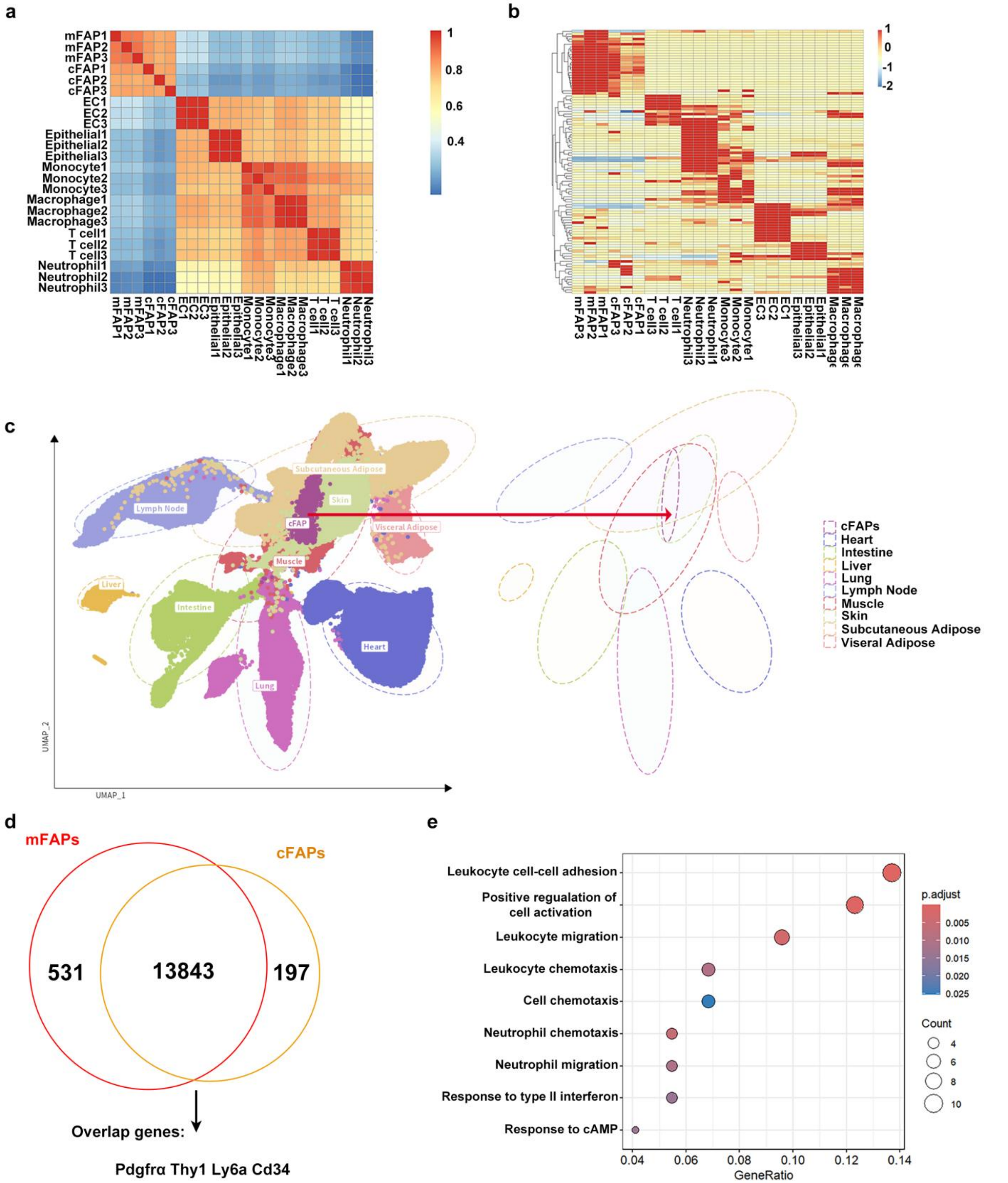

Supplementary Figure S7. Characteristics of circulating FAPs.

(a) Sample clustering based on RNA-seq data from muscle FAPs (mFAPs), circulating FAPs (cFAPs), endothelial cells (ECs), epithelial cells, monocyte, macrophage, T cells and neutrophil.

(b) Heatmap showing the gene expression patterns of different cell types.

(c) UMAP displaying the similarity between cFAPs and fibroblasts derived from different tissues.

(d) Venn diagram showing the overlap and different expressed genes in cFAPs or mFAPs.

(e) GO enrichment analysis of differentially expressed genes between cFAPs and mFAPs.

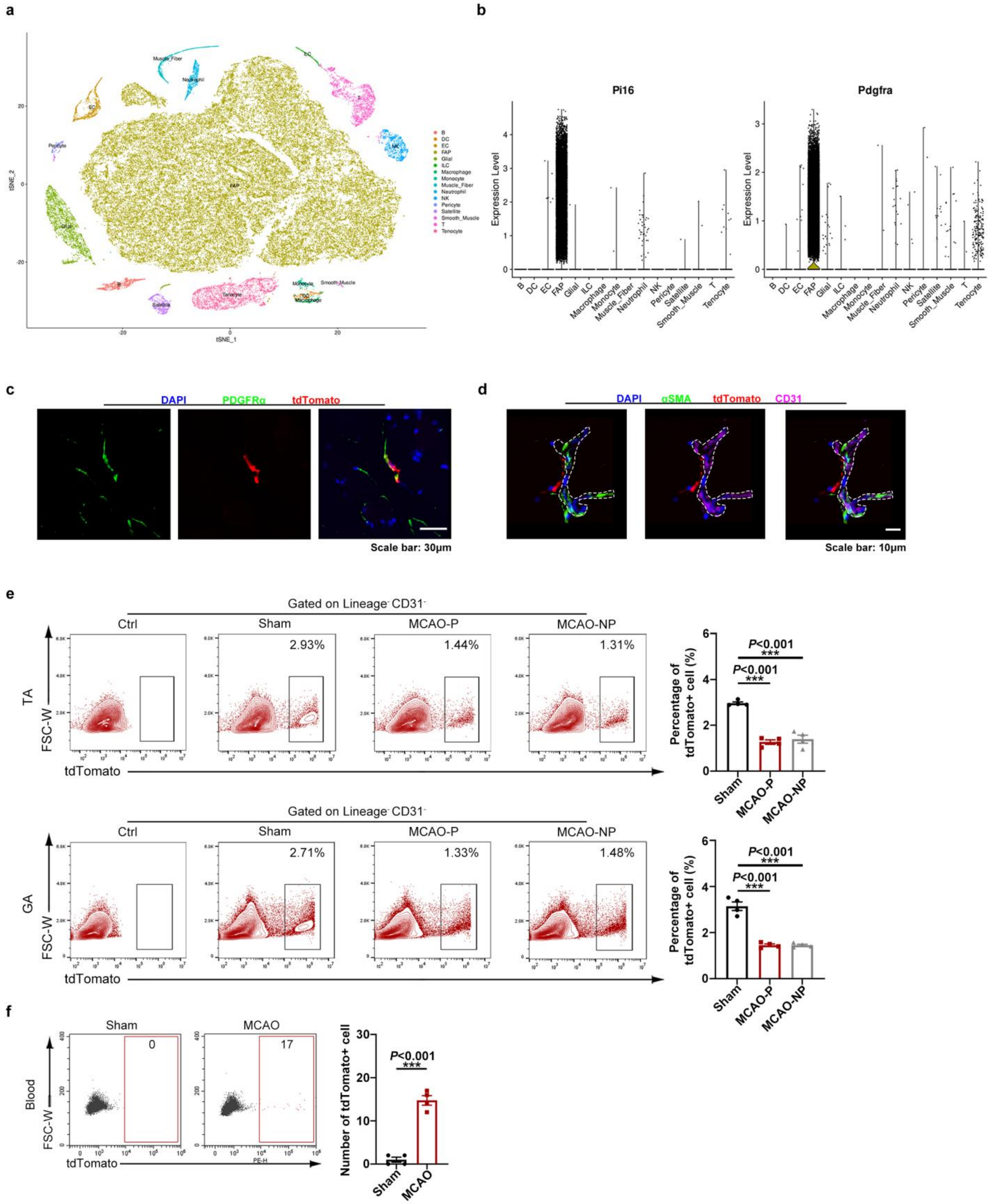

Supplementary Figure S8. Stroke mobilizes PI16^{+} FAPs to egress from skeletal muscle into circulation.

(a) Single-cell atlas of different cell types in skeletal muscle tissue.

(b) The expression distribution of Pdgfra and Pi16 in skeletal muscle was shown as violin plots.
(c-d) Representative confocal images of double immunostaining for PDGFRα with CD31 (c) and αSMA (d) in TA muscle sections of *Pi16-creER; Rosa26-tdTomato* mice.
(e) FACS analysis showing reduced tdTomato-labeled $PI16^+$ FAPs in the hindlimb after MCAO. n=4.
(f) Representative FACS panels showing increased number of tdTomato-labeled $PI16^+$ FAPs in peripheral blood 3 days after MCAO. n=4.

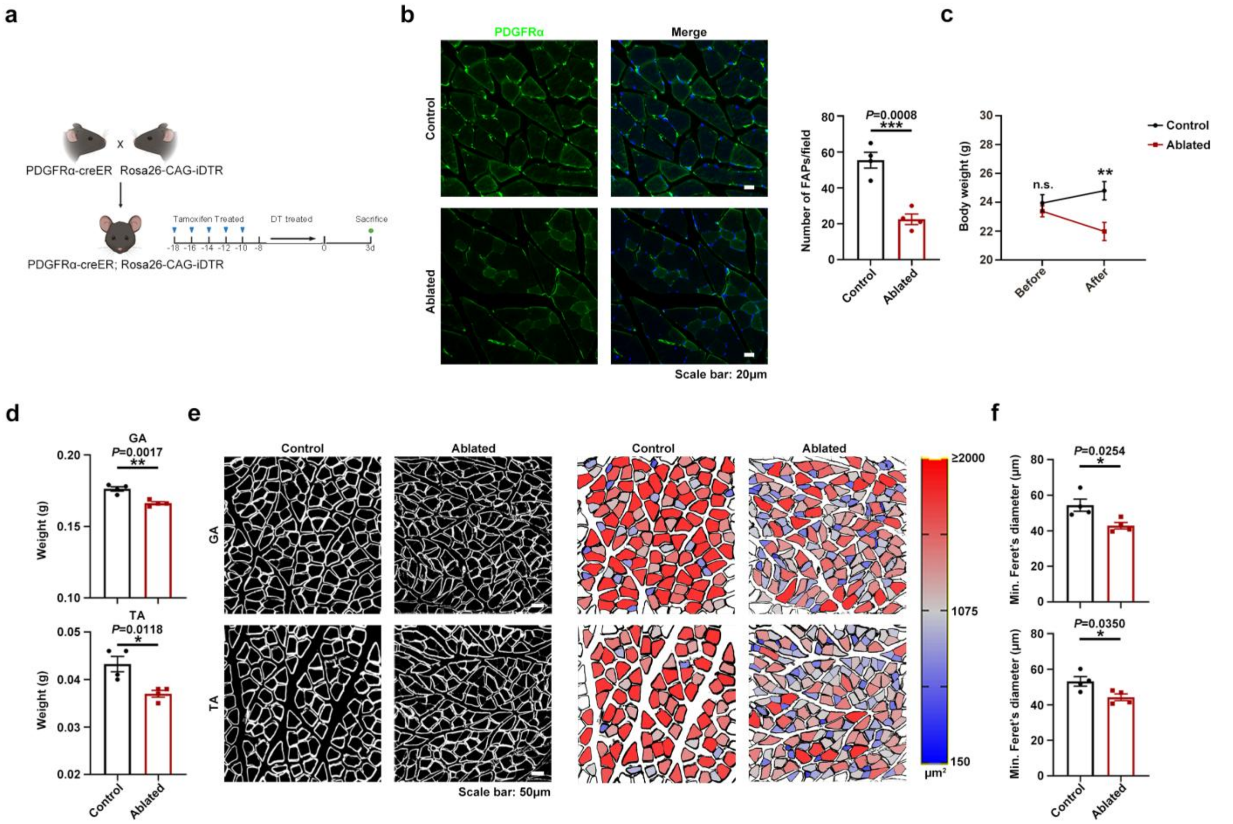

Supplementary Figure S9. Depletion of PDGFRα$^+$ cells leads to body weight loss and muscle atrophy.

(a) Schematic diagram of PDGFRα$^+$ cells ablation.

(b) The efficiency of Cre-dependent ablation of FAPs in TA muscle from control or *PDGFRα-CreER; Rosa26-iDTR* mice. Scale bar: 20μm.

(c-d) Assessment of body weight (c) and muscle weight (d) 3 days after FAPs ablation. n=4.

(e) Representative immunostaining images of TA and GA muscle for Laminin. Scale bar: 50 μm.

(f) Quantitation of the minimal Feret's diameter of myofiber. n=4.

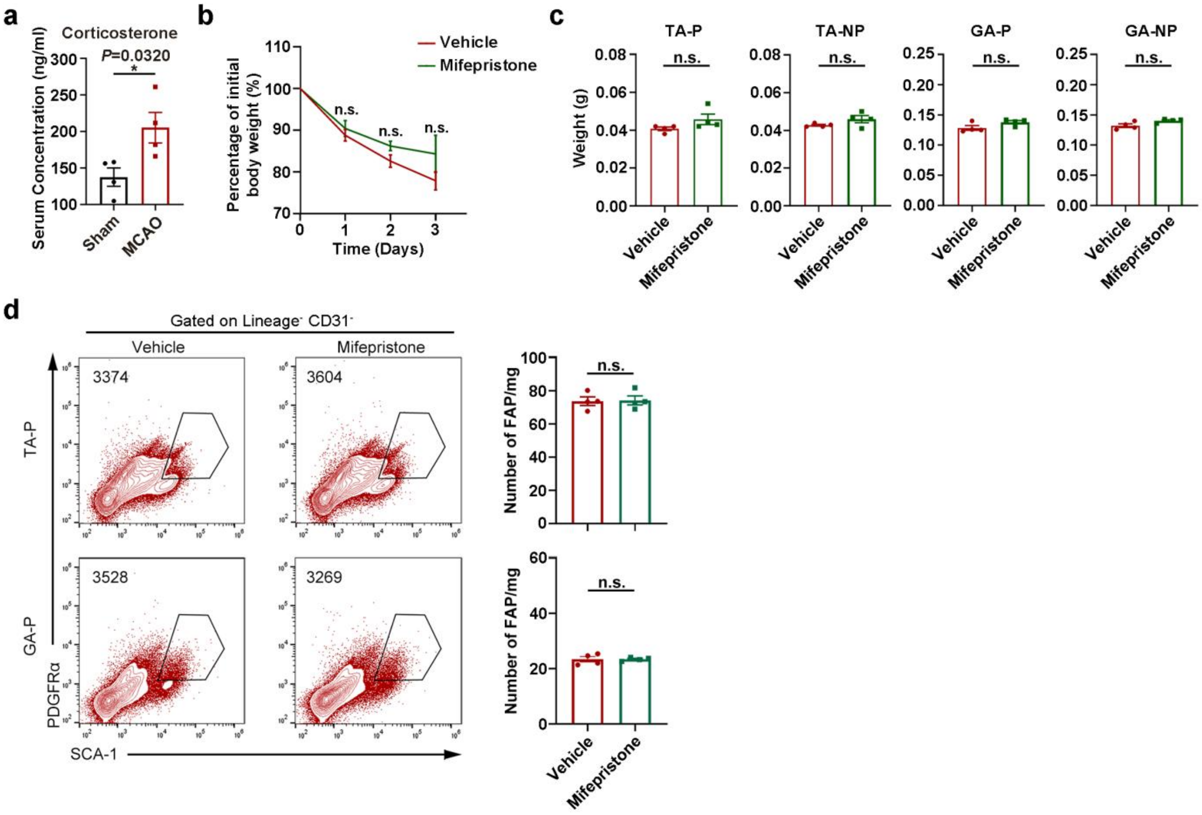

Supplementary Figure S10. GR mifepristone antagonist fails to rescue FAP loss and subsequent sarcopenia.

(a) ELISA assay was applied to measure the concentration of serum corticosterone at 3 days after MCAO. n=4.

(b-c) Quantification of body weight (b) and muscle weight (c) after 3-day mifepristone treatment in MCAO mice. n=4.

(d) FACS panels showed the number of PDGFRα^{+} SCA-1^{+} FAPs in paretic limb muscles. n=4.

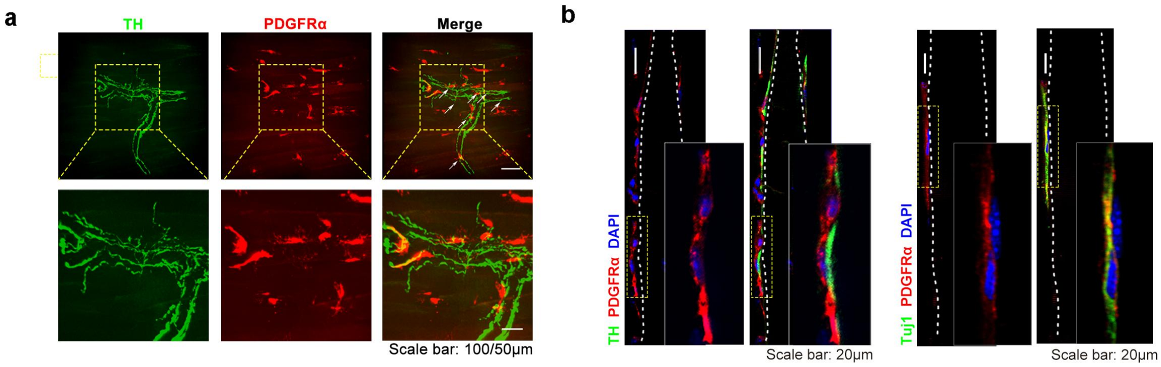

Supplementary Figure S11. Colocalization analysis of TH^{+} sympathetic fibers with PDGFRα^{+} FAPs in cross and longitudinal sections of TA muscle.

Scale bar: 100/50 μm (a), 20 μm (b).

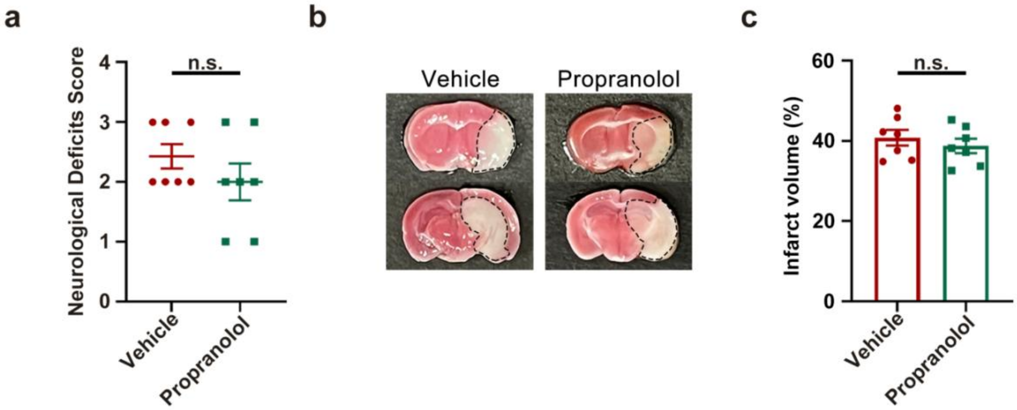

Supplementary Figure S12. Systemic propranolol treatment exerts no protective impacts on neurological scores or infarct volumes of MCAO mice.

(a) Neurological deficits scores were evaluated in vehicle and propranolol-treated MCAO mice respectively. n=7.

(b-c) Infarct volumes analysis with TTC staining of coronal brain sections at 3 days after MCAO. n=7.

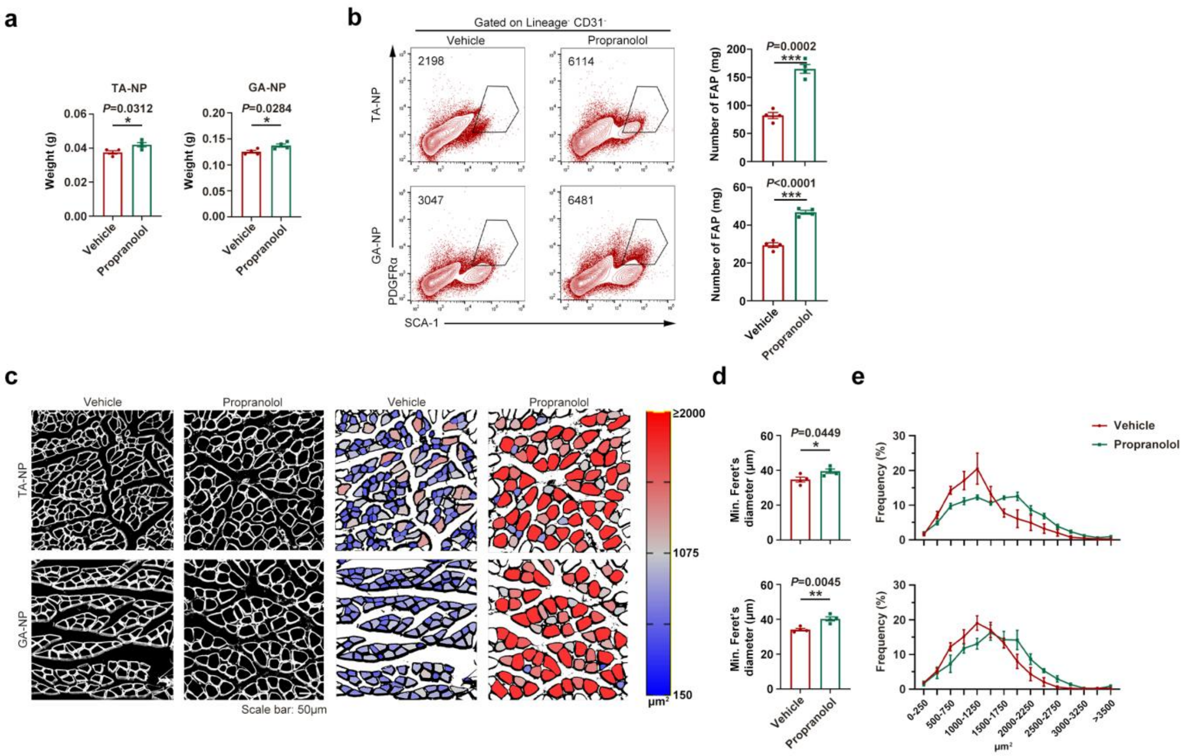

Supplementary Figure S13. β-blocker propranolol restores FAPs and muscle mass in non-paretic limb of MCAO mice.

(a) Quantification of muscle weight in non-paretic limbs after 3-day propranolol treatment. n=4.

(b) FACS panels showed the number of PDGFRα$^+$ SCA-1$^+$ FAPs in non-paretic limb muscles. n=4.

(c) Histological assessment of TA and GA muscle from the non-paretic side of propranolol-treated mice and vehicle-treated controls. Scale bar: 50 μm.

(d-e) Quantification of minimal Feret's diameter (d) and myofiber cross sectional area (CSA) frequency (e). n=4.

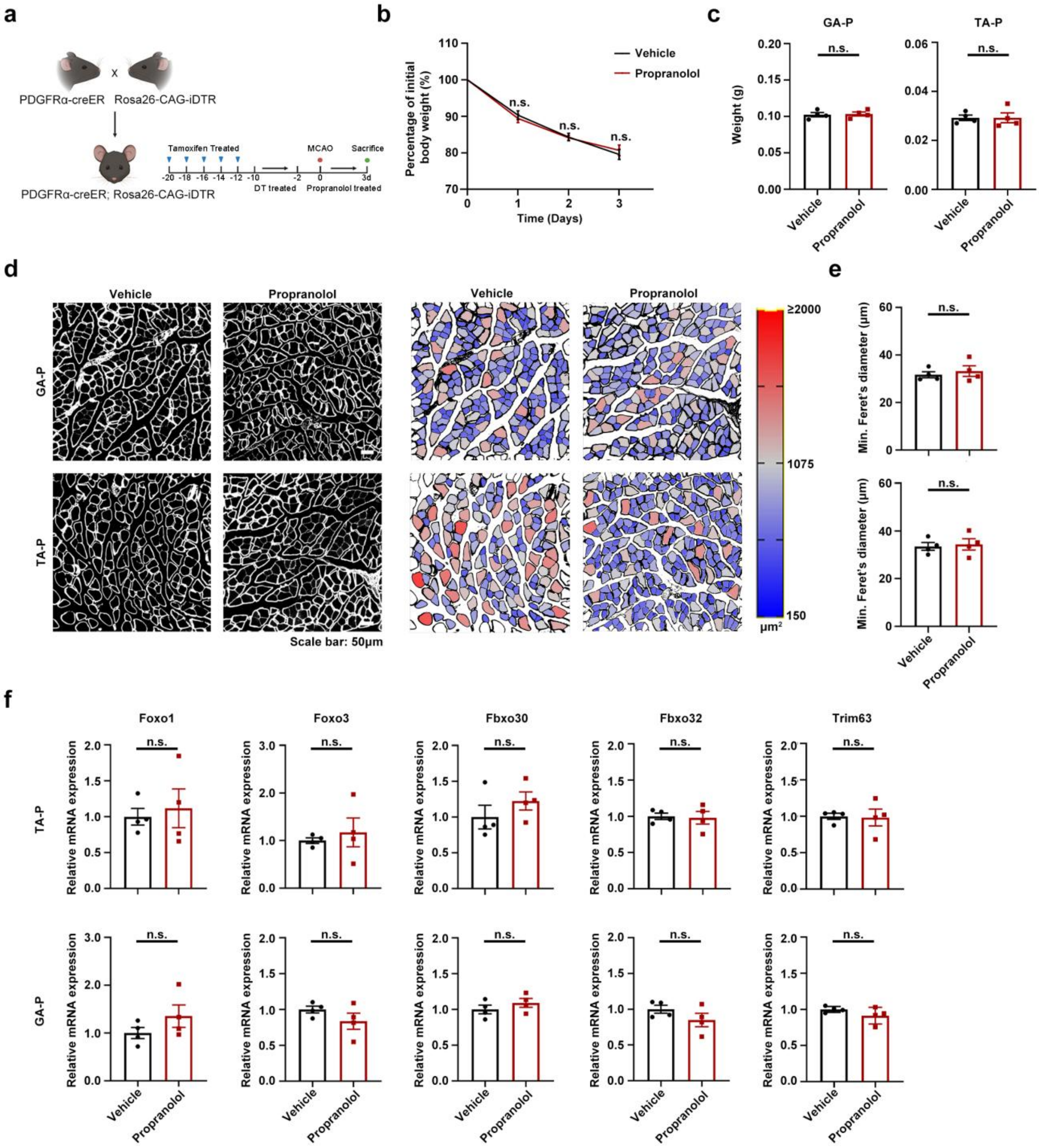

Supplementary Figure S14. Propranolol combats stroke-related sarcopenia in a FAP-dependent mechanism.

(a) Schematic diagram showing the propranolol treatment strategy for *PDGFRα-CreER; Rosa26-iDTR* mice.

(b-c) Quantification of body weight (b) and muscle weight (c) after 3-day propranolol treatment in stroke *PDGFRα-CreER; Rosa26-iDTR* mice. n=4.

(d) Histological assessment of TA and GA muscle from the paretic side of vehicle or propranolol-treated *PDGFRα-CreER; Rosa26-iDTR* mice 3 days after stroke. Scale bar: 50 μm.
(e) Minimal Feret's diameter in TA and GA muscle from the paretic side. n=4.
(f) Muscle atrophy gene expression was measured by qRT-PCR in vehicle- or propranolol-treated *PDGFRα-CreER; Rosa26-iDTR* mice 3 days after stroke. n=4.

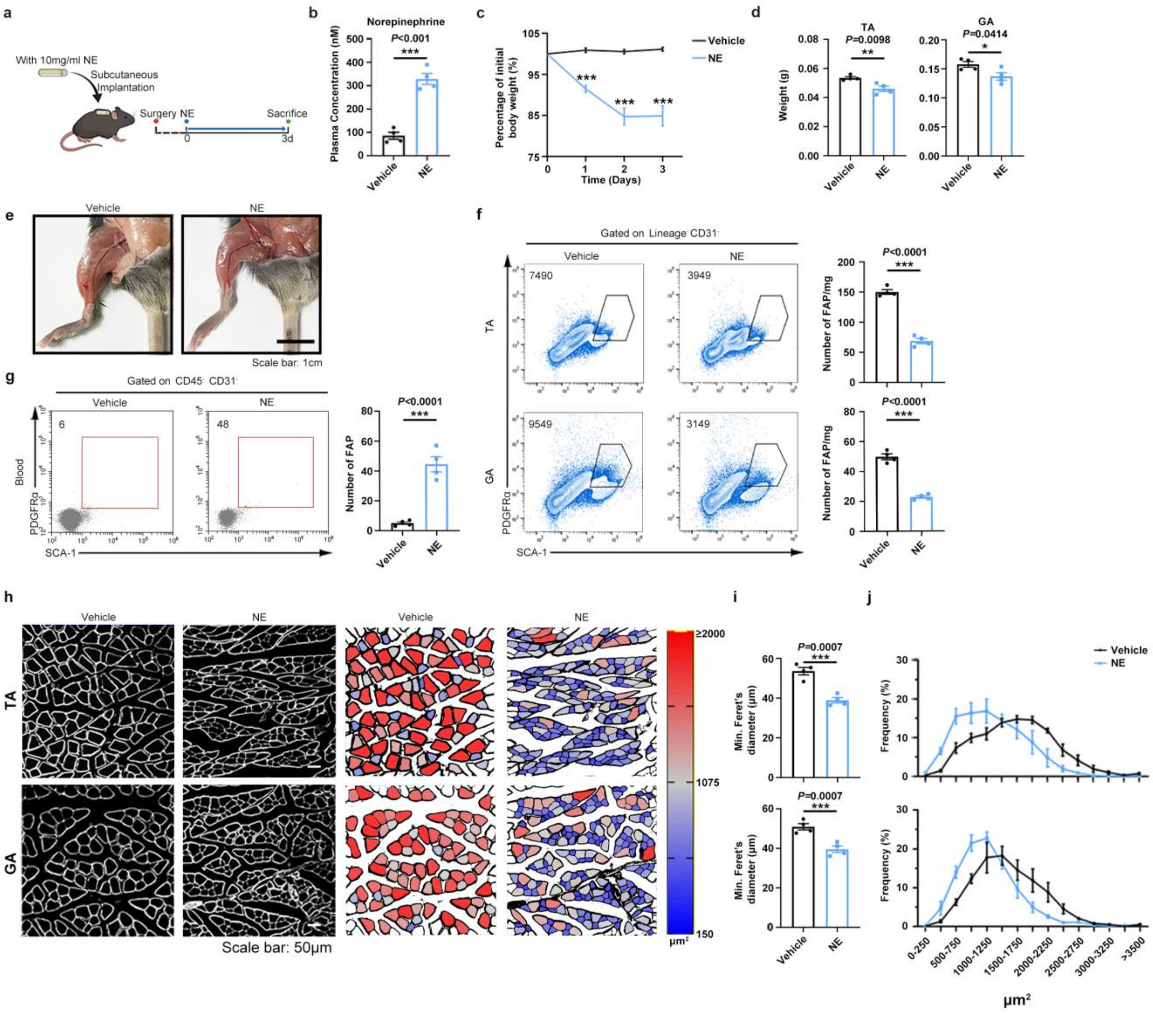

Supplementary Figure S15. Continuous release of norepinephrine contributes to FAP outflow from skeletal muscle.

(a) Schematic diagram of continuous NE delivering by osmotic pumps.

(b) ELISA assay was applied to measure the concentration of plasma norepinephrine (NE) at 3 days after osmotic pumps implantation. n=4.

(c-d) Quantification of body weight (c) and muscle weight (d) after 3-day norepinephrine or vehicle treatment in C57BL/6 mice. n=4.

(e) Representative photographs of lower limbs from norepinephrine-treated or vehicle-treated mice. Scale bar: 1cm.

(f-g) The numbers of PDGFRα$^+$ SCA-1$^+$ FAPs in limb muscles (f) and the peripheral blood (g) were quantified by FACS in vehicle control and norepinephrine-treated mice. n=4.

(h) Representative muscle cross sections stained with laminin for the CSA quantification. Scale bar: 50 μm.

(i-j) Quantitative of myofiber CSA (i) and minimal Feret's diameter (j). n=4.

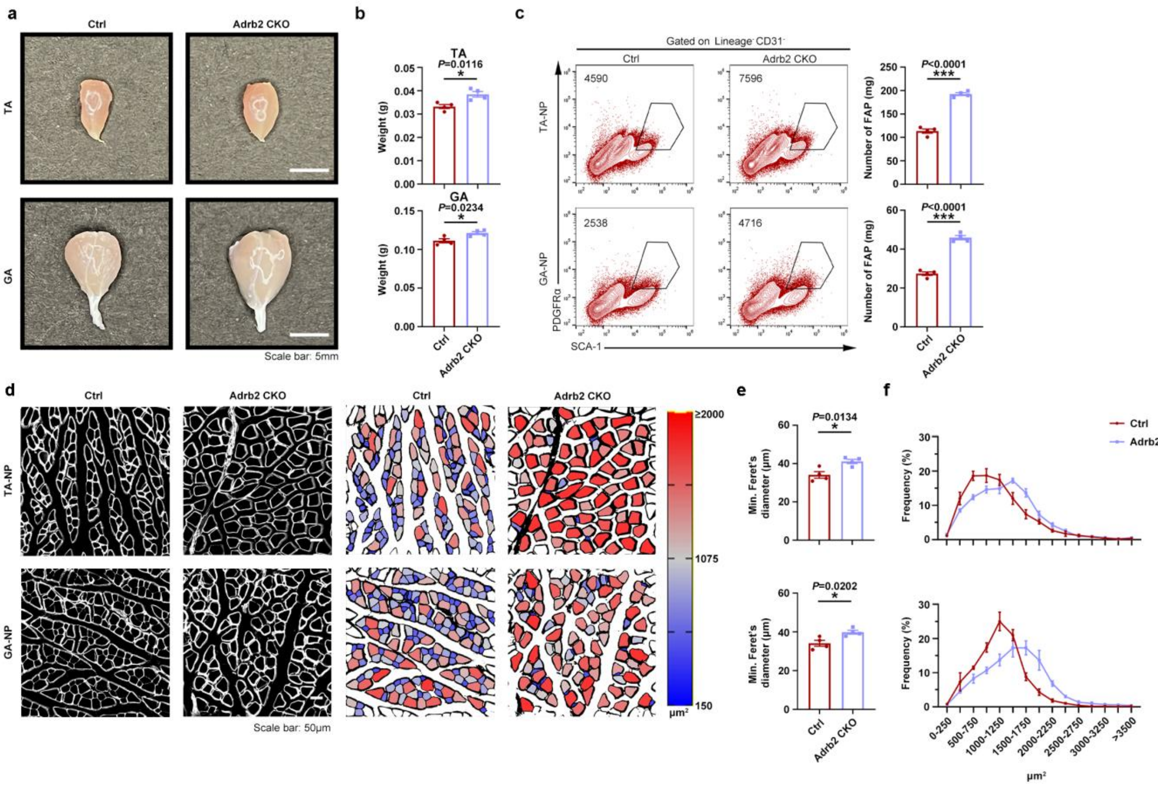

Supplementary Figure S16. Stroke-related sarcopenia is markedly blunted in the non-paretic limb muscle of Adrb2 cKO mice.

(a) Representative photographs of non-paretic TA and GA from control and *Adrb2* cKO mice at 3 days after MCAO. Scale bar: 1 cm.

(b) Non-paretic TA and GA muscle weight normalized by initial body weight of littermate sibling controls (*PDGFRα-CreER*$^{-/-}$*; Adrb2*$^{fl/fl}$) and *Adrb2* cKO mice at 3 days after MCAO. n=4.

(c) FACS profiles for assessing the number of PDGFRα$^+$ SCA-1$^+$ FAPs in non-paretic limb muscles of control and *Adrb2* cKO mice at 3 days after MCAO surgery. n=4.

(d-f) Detection of myofiber size by Laminin staining of muscle cross sections (d). The graphs indicated that paretic muscle Min. Feret's diameter (e) and CSA (f) markedly increased in *Adrb2* cKO mice compared to control littermates. Scale bar: 50 μm. n=4.

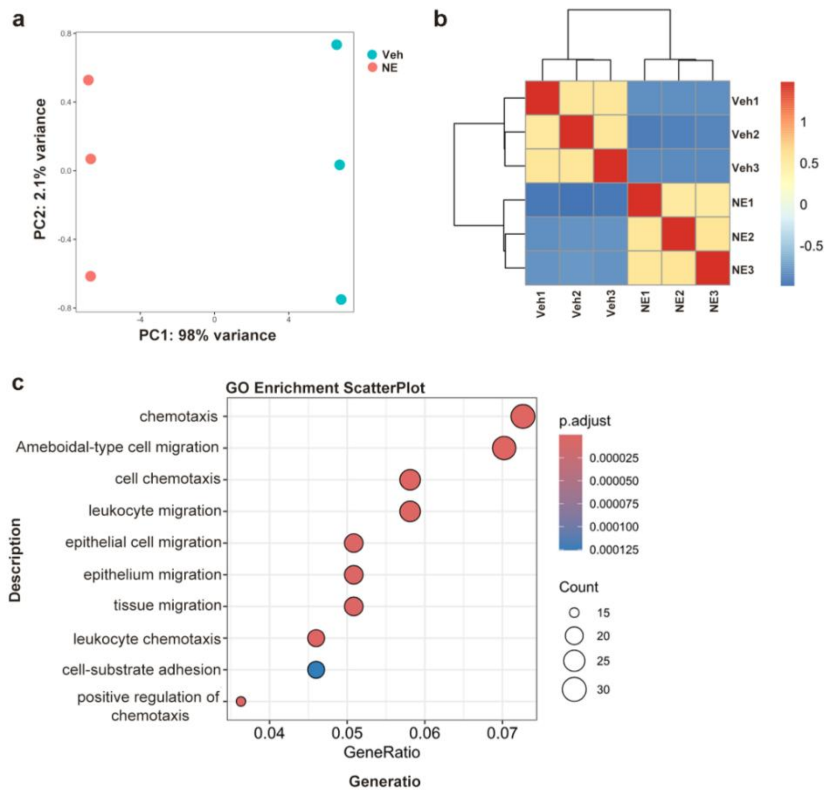

Supplementary Figure S17. Bulk RNA-seq analysis of FAPs from NE-treated mice compared to vehicle-treated mice.

(a) Representative principal component analysis of FAPs isolated from vehicle- and NE-treated mice. Vehicle n=3, NE n=3.

(b) Correlation matrix heatmap displaying different transcripts between FAPs from vehicle- and NE-treated mice. Vehicle n=3, NE n=3.

(c) GO enrichment analysis of differentially expressed genes in FAPs from NE-treated mice versus vehicle-treated controls.

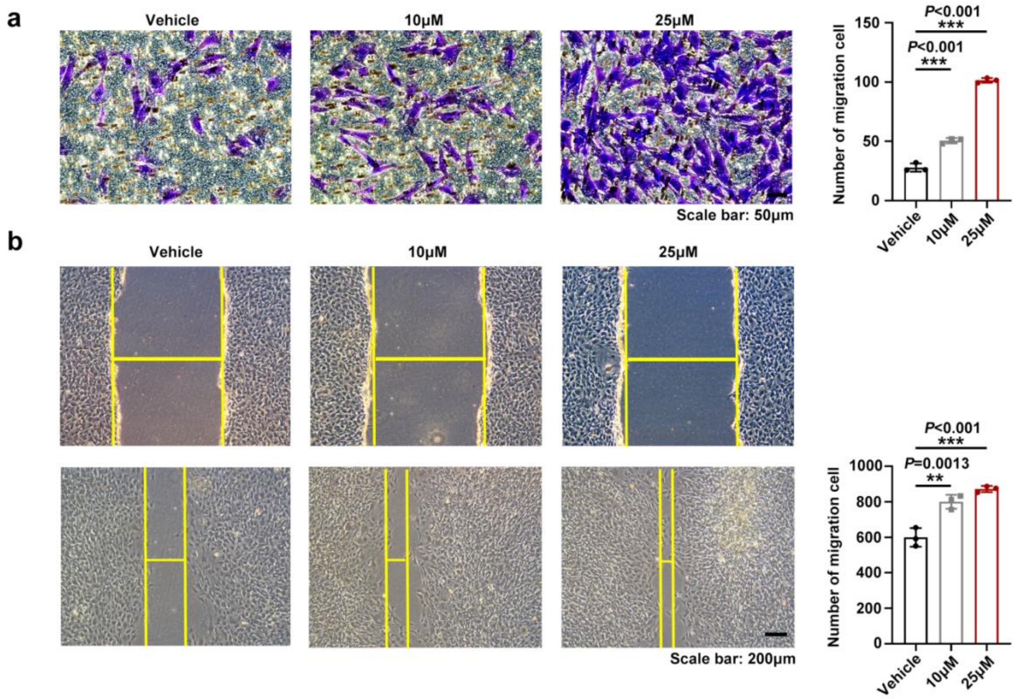

Supplementary Figure S18. NE administration facilitates FAPs migration *in vitro*.

(a-b) Assessment of migratory capacity of FAPs in response to NE treatment using transwell (a) and scratch wound healing assay (b). n=3. Scale bar: 50/200μm.

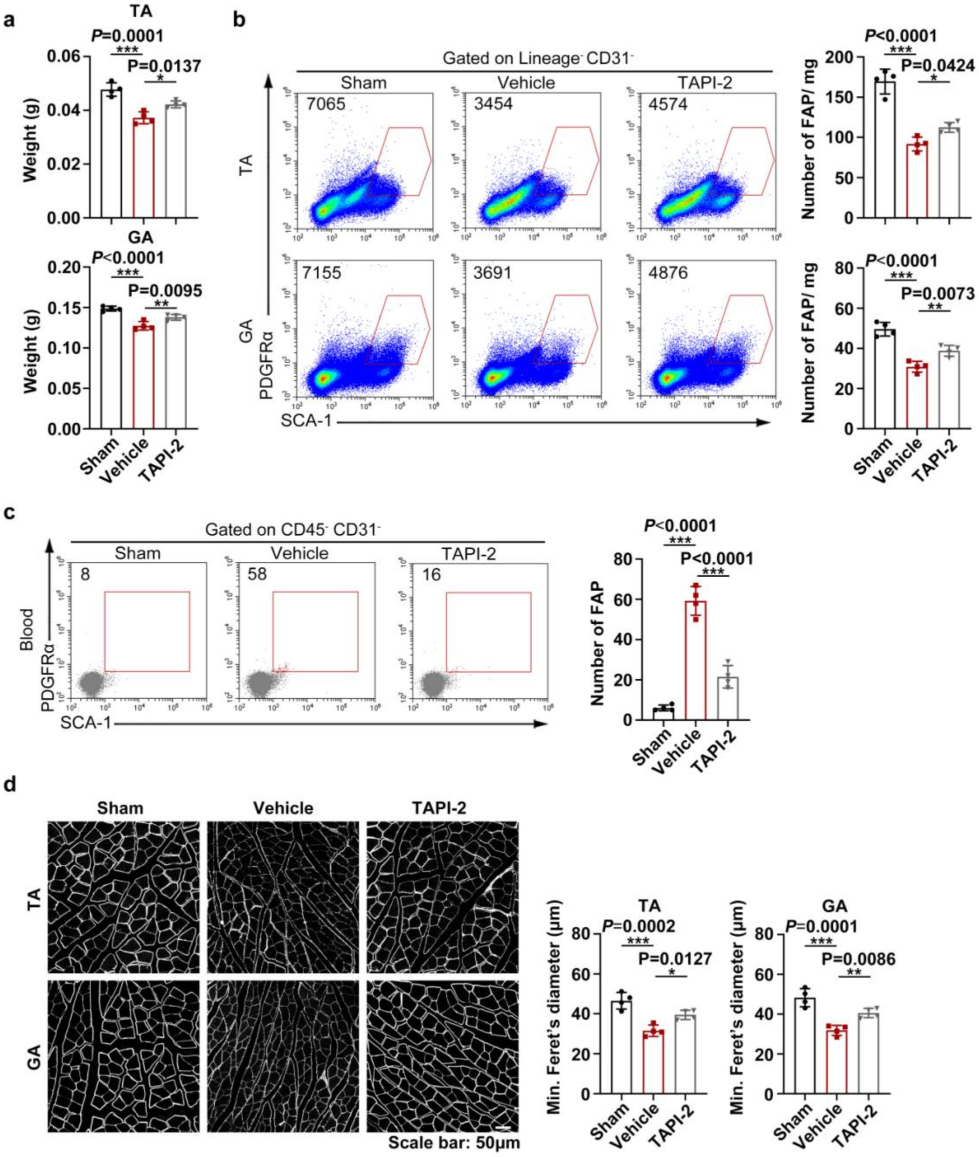

Supplementary Figure S19. TAPI-2 restores FAPs and muscle mass in MCAO mice.

(a) Quantification of absolute muscle weight after 3-day TAPI-2 treatment in MCAO mice. n=4.

(b-c) FACS panels showed the number of PDGFRα$^+$ SCA-1$^+$ FAPs in paretic limb muscles (B) and the peripheral blood (C). n=4.

(d) Quantitative of myofiber minimal Feret's diameter of TA and GA muscle. n=4. Scale bar: 50μm.

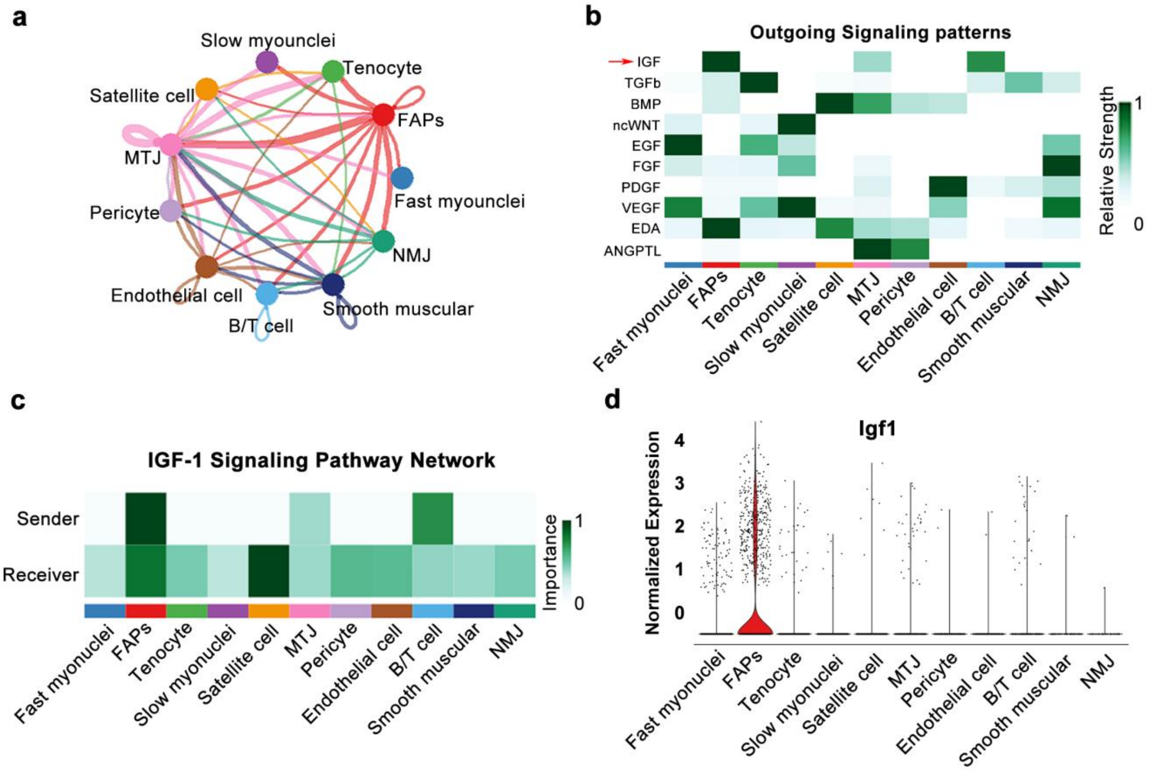

Supplementary Figure S20. CellChat analysis based on single-nucleus RNA-seq data from murine skeletal muscle.

(a) Circle plot showing ligand-receptor pairs between any two cell clusters in skeletal muscle.

(b) Outgoing signaling patterns in each cell cluster was shown as a heatmap.

(c) Heatmap of relative contribution of each cell subset in IGF-1 signaling pathway.

(d) Violin plots of *Igf1* expression in distinct cell populations based on murine skeletal muscle single-nucleus RNA-seq data.

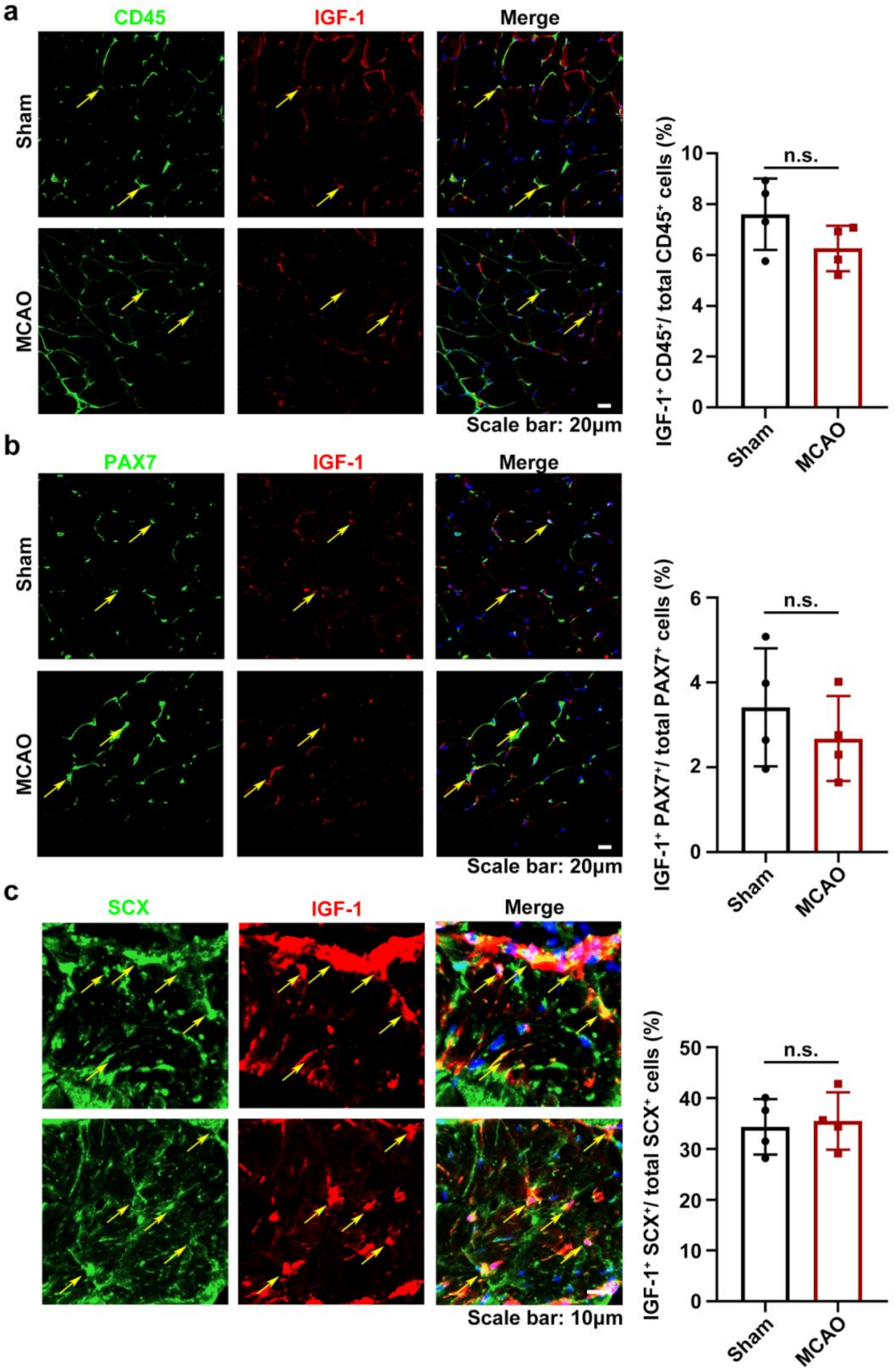

Supplementary Figure S21. Immunofluorescence staining for the detection of IGF-1^+ cells in immune cells (a), MuSCs (b) and tendon cells (c) of skeletal muscle tissue after stroke. Scale bar: 20/5μm.

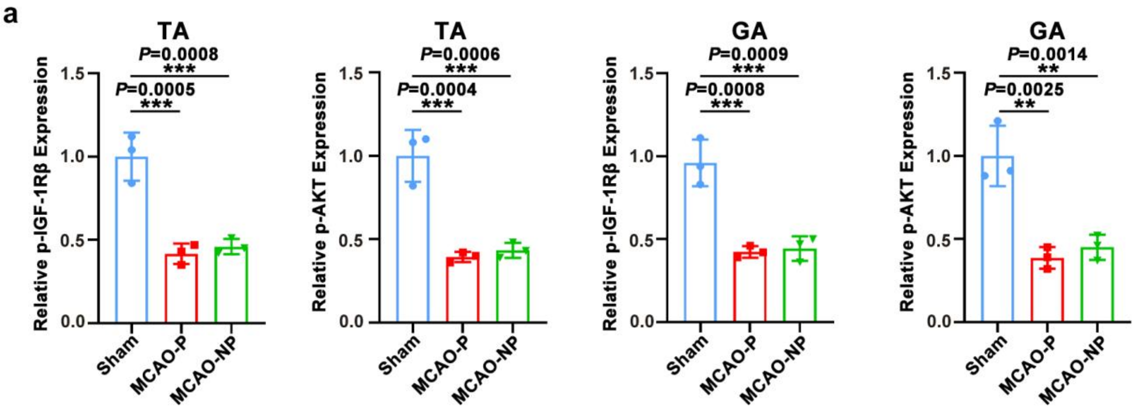

Supplementary Figure S22. Quantification of Western Blot analysis for the phospho-IGF-1Rβ and phospho-AKT levels in both TA and GA muscle at 3 days after stroke. n=3.

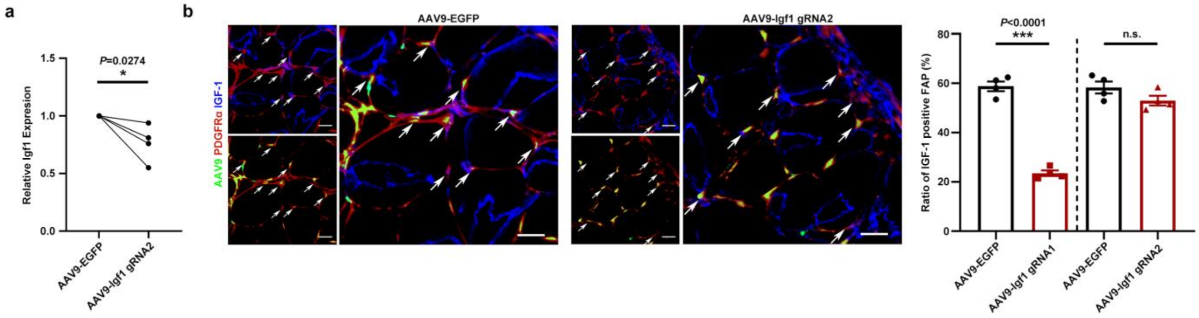

Supplementary Figure S23. The editing efficiency of gRNA-2 targeting *Igf1*.

(a-b) The mRNA expression levels (a) and protein abundance (b) of IGF-1 in AAV9-EGFP or AAV9-*Igf1* gRNA-injected TA muscle. Scale bar: 20 μm. n=3.

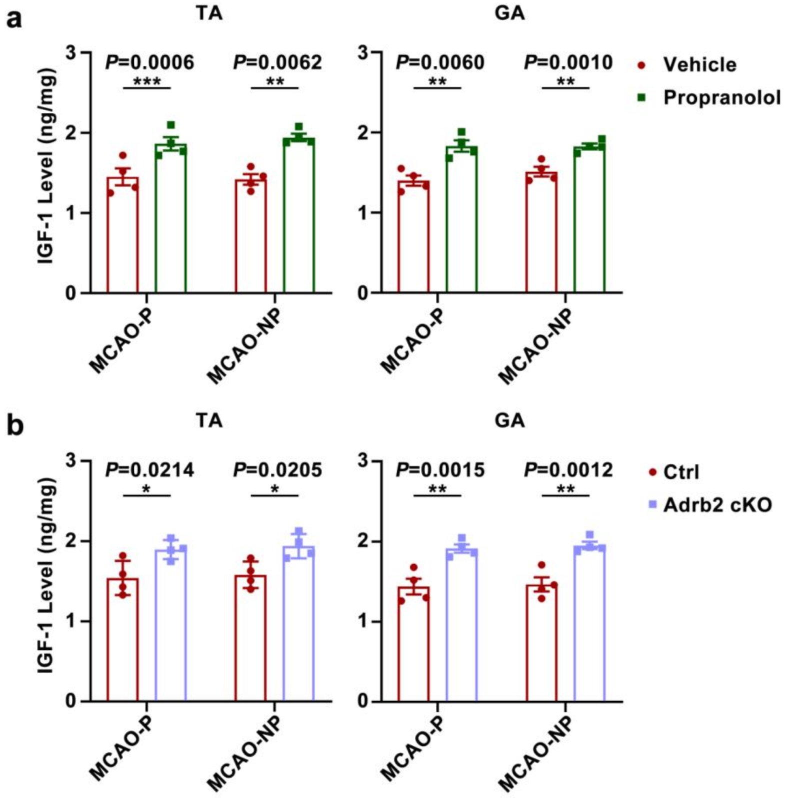

Supplementary Figure S24. Propranolol treatment (a) and *Adrb2* knockout (b) partially restored IGF-1 levels in the TA and GA muscle.

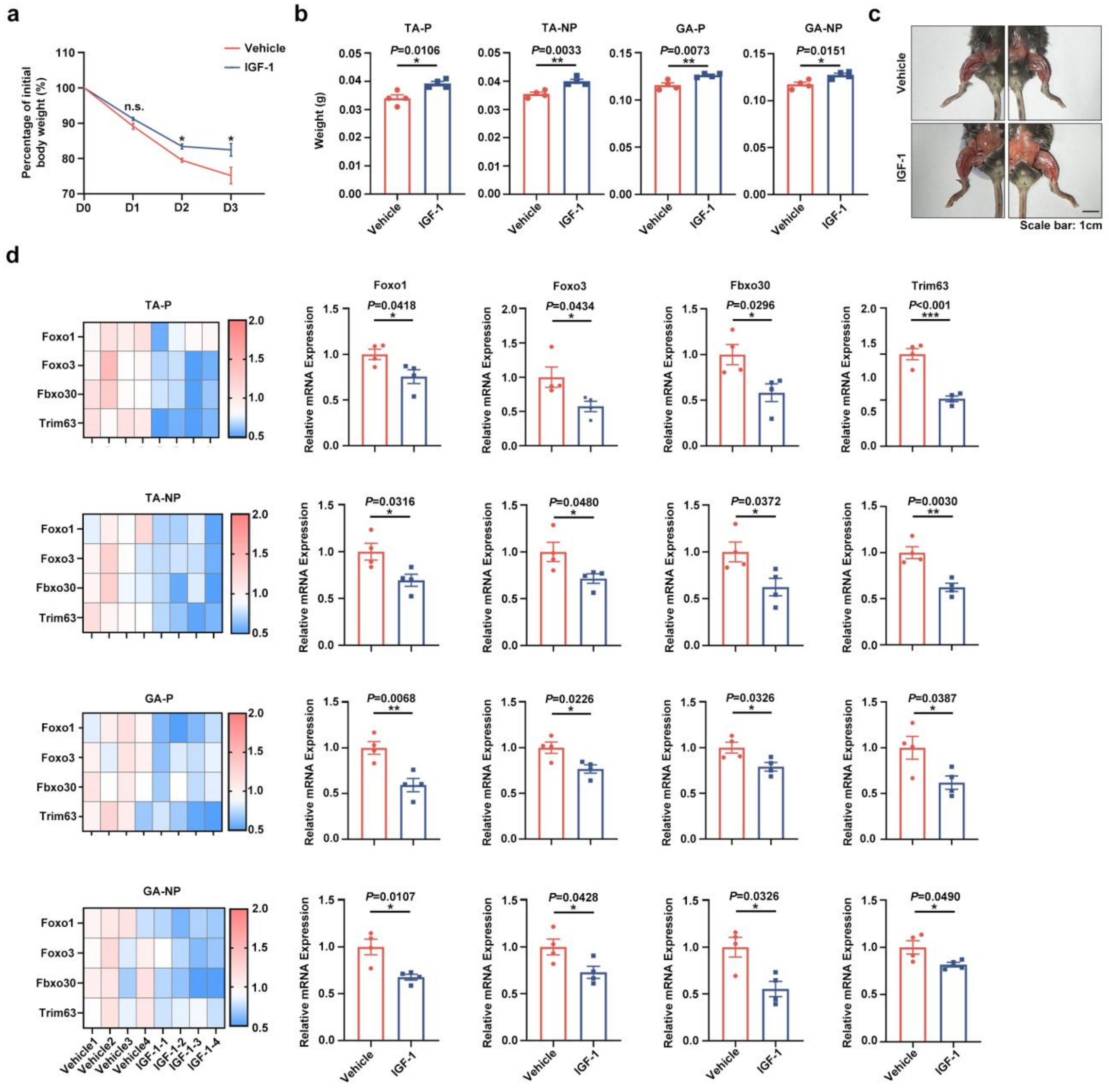

Supplementary Figure S25. IGF-1 treatment restores muscle mass in MCAO mice.

(a-b) Quantification of body weight (a) and absolute muscle weight (b) after systemic injection of IGF-1 for consecutive 3 days in MCAO mice. n=4.

(c) Representative photographs of lower limbs from vehicle or IGF-1-treated MCAO mice at 3 days after stroke. Scale bar: 1 cm.

(d) Atrophy genes expression measured by qRT-PCR of lower limbs muscle in vehicle- and IGF-1-treated mice. n=4.
